# A two-step DNA barcoding approach for delimiting moth species: moths of Dongling Mountain (Beijing, China) as a case study

**DOI:** 10.1038/s41598-018-32123-9

**Published:** 2018-09-24

**Authors:** Qian Jin, Xi-Min Hu, Hui-Lin Han, Fen Chen, Wei-Jia Cai, Qian-Qian Ruan, Bo Liu, Gui-Jie Luo, Hao Wang, Xu Liu, Robert D. Ward, Chun-Sheng Wu, John-James Wilson, Ai-Bing Zhang

**Affiliations:** 1 Suqian Institute of Agricultural Sciences, Jiangsu Academy of Agricultural Sciences, Suqian, Jiangsu 223800 China; 2 College of Life Sciences, Capital Normal University, Beijing, 100048 China; 3 School of Forestry, Experiment Center, Northeast Forestry University, Haerbin, 150040 China; 4 CSIRO National Research Collections Australia, GPO Box 1538, Hobart, Tasmania 7001 Australia; 5 Institute of Zoology, Chinese Academy of Sciences, 1 Beichen West Road, Chaoyang District, Beijing, 100101 China; 6 School of Applied Sciences, Faculty of Computing, Engineering and Science, University of South Wales, Pontypridd, CF37 4AT United Kingdom; 7 Department of Microbiology and Parasitology, Faculty of Medical Science, Naresuan University, Phitsanulok, 65000 Thailand

## Abstract

DNA barcoding, based on a fragment of cytochrome c oxidase I (COI) mtDNA, is as an effective molecular tool for identification, discovery, and biodiversity assessment for most animals. However, multiple gene markers coupled with more sophisticated analytical approaches may be necessary to clarify species boundaries in cases of cryptic diversity or morphological plasticity. Using 339 moths collected from mountains surrounding Beijing, China, we tested a pipeline consisting of two steps: (1) rapid morphospecies sorting and screening of the investigated fauna with standard COI barcoding approaches; (2) additional analyses with multiple molecular markers for those specimens whose morphospecies and COI barcode grouping were incongruent. In step 1, 124 morphospecies were delimited into 116 barcode units, with 90% of the conflicts being associated with specimens identified to the genus *Hypena*. In step 2, 55 individuals representing all 12 *Hypena* morphospecies were analysed using COI, COII, 28S, EF-1a, Wgl sequences or their combinations with the BPP (Bayesian Phylogenetics and Phylogeography) multigene species delimitation method. The multigene analyses supported the delimitation of 5 species, consistent with the COI analysis. We conclude that a two-step barcoding analysis pipeline is able to rapidly characterize insect biodiversity and help to elucidate species boundaries for taxonomic complexes without jeopardizing overall project efficiency by substantially increasing analytical costs.

## Introduction

DNA barcoding – the sequencing of a short, standard genetic marker from unknown specimens coupled with analyses of sequence divergences^1,2^ – has been shown to be a practical tool for species identification and biodiversity assessment^3–5^. DNA barcodes can also provide information for clarifying species boundaries, especially in taxa that are poorly studied, species-rich or whose morphological characters are limited^6,7^. Consequently, many cryptic species have been uncovered through DNA barcoding, increasing the number of recognized species across many taxa^5,8,9^. For example, in north-western Costa Rica, Hebert and colleagues^8^ uncovered ten cryptic species of butterflies collectively known as *Astraptes fulgerator*, while Smith and colleagues^10^ discovered 12 cryptic species in a genus of parasitoid flies (*Belvosia*). More recently, Janzen and colleagues discovered that a group of widespread neotropical skipper butterflies, collectively known as *Udranomia kikkawai* (Weeks), comprised a complex of three species^11^. However, some studies have indicated that DNA barcoding can overestimate species richness^12,13^. Brower^14^ concluded that there were only three to seven cryptic species of *Astraptes fulgerator* rather than the ten suggested by Hebert *et al*.^8^, while Dasmahapatra *et al*.^15^ concluded that only one of four ‘cryptic species’ in the butterfly genus *Mechanitis* was biologically meaningful. Despite these disagreements, which may reflect disparities in notions of what constitutes a species and how they are recognized, a general consensus has emerged that standard COI barcodes can meet the needs of much conventional species identification and delimitation^2,16–22^.

Nonetheless, there are potential problems encountered when using mitochondrial DNA to infer species boundaries, arising from: its characteristic maternal inheritance; difficulties caused by hybridization or introgression; sex-biased gene flow; cytoplasmic incompatibility-inducing symbionts (Wolbachia infecting 66% of all insect species^23^); horizontal gene transfer^24^; nuclear copies of mitochondrial genes^25,26^ and “reticulate” evolutionary phenomena in lineages. Such factors may account for underestimates or overestimates of species richness^3,25^. Clearly, a mitochondrial single-locus approach can occasionally be problematic for accurate species delimitation^27,28^ and, especially for taxonomically contentious groups, “independent” nuclear genes may be needed as supplementary markers to support any conclusions^11^.

For molecular species delimitation, once DNA sequences have been obtained, analyses are necessary to partition sequence variation into intraspecific and interspecific divergences^2^. Hebert and colleagues^29^ initially proposed a standard sequence threshold of 10 times the mean intraspecific divergence to delimit animal species. Subsequently, more sophisticated statistical approaches were proposed, for example jMOTU^30^ and Automatic Barcode Gap Discovery (ABGD)^31^. Pons and colleagues^32^ proposed delimiting species using a mixed model combining a coalescent population model with a Yule model of speciation; the general mixed Yule coalescent model (GMYC) has become one of the most popular approaches for single-locus species delimitation. By far the most popular multi-locus species delimitation method is Bayesian Phylogenetics and Phylogeography (BPP)^33^, which delimits species using a reversible jump Markov chain Monte Carlo (rjMCMC) algorithm. The BPP method is grounded on the multispecies coalescent model and calculates the posterior probabilities of competing models that contain more, or fewer, lineages.

The order Lepidoptera is the second largest insect order, comprising about 174,250 species in 126 families and 46 superfamilies^34^. The order represents the largest radiation of herbivorous animals in the history of our planet and lepidopterans play vital roles throughout ecosystems. Many lepidopteran larvae are major pests of crops and forests^35^, and their control and monitoring requires accurate species identification and delimitation, a challenge considering the few taxonomic specialists and large amounts of undescribed and cryptic biodiversity^36–38^. As the second richest noctuid genus following *Euxoa* Hübner, a pertinent example is the genus *Hypena* (Lepidoptera, Noctuidae s.l.), which includes many significant agricultural and forest pests^39–42^. Delimitation of *Hypena* species relies to a considerable extent upon the dissection and examination of genitalia, but dissections are difficult to prepare due to the flabby consistency and oily appearance of the valves (noted as a “physiological synapomorphy” by Lödl^40^). These problems make morphological species identification difficult^43^, and an alternative convenient and reliable approach is highly desirable.

We collected moths (Lepidoptera), including representatives of *Hypena*, from Beijing, China, to investigate the role of DNA barcoding for species delimitation. We deployed a two-step DNA barcoding approach for delimiting moth species: (1) rapid sorting into morphospecies and molecular operational taxonomic units (MOTU) with a standard COI protocol, and (2) further analyses with additional molecular markers for those specimens whose single-locus results conflict with morphospecies grouping, and geometric morphometric analyses to probe morphological wing variation within the conflicting groupings.

## Results

### Step 1: Standard COI protocol for MOTU delimitation

**Figure 1:**
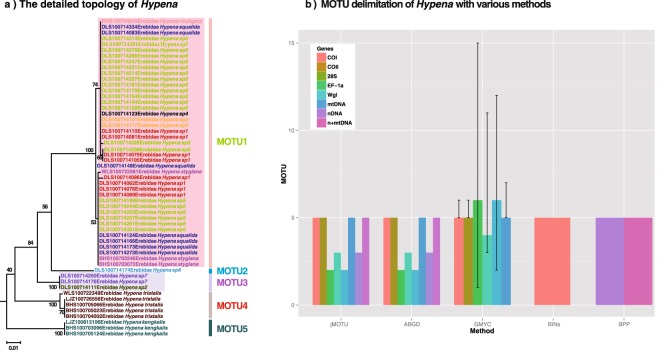
(**a**) The detailed topology of *Hypena* sequences. Different font colors indicates different morphospecies. Vertical line represents GMYC MOTU. (**b**) MOTU delimitation of *Hypena* under five approaches.

351 moth (Lepidoptera) specimens from 124 morphospecies (10 families and 84 genera) showed a sequencing success rate of 100% for the COI barcode. Almost all morphospecies possessed a cluster of unique sequences and there were 116 morphospecies clusters with >95% bootstrap values (see Supplementary Fig. S1). The exceptions (accounting for 7.5% of all morphospecies) were the noctuid species *Acosmetia chinensis* and *A*. *biguttula* and the taxonomically difficult noctuid genus *Hypena* (sp1, sp3, sp4, sp5, *H*. *squalida*, *H*. *stygiana* and *H*. *rivuligera* share haplotypes with one another while sp2 and sp7 share an identical haplotype; Fig. 1) (see Supplementary Fig. S1).

**Figure 2:**
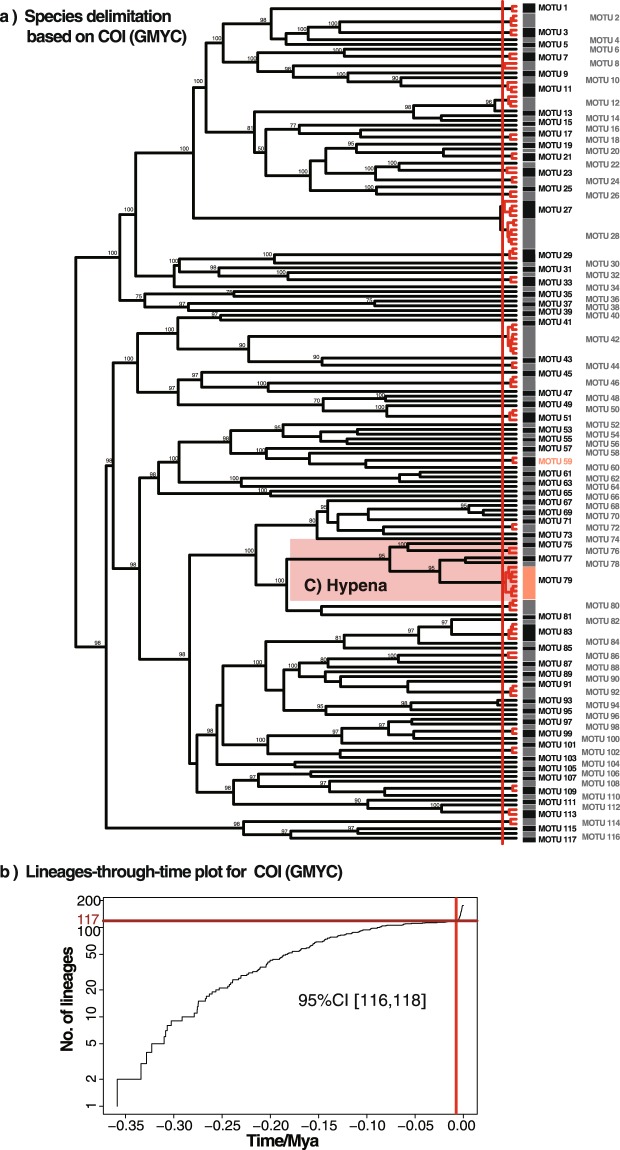
GMYC analysis of the 176 COI unique haplotypes for all 351 moth specimens. The red vertical lines on the ultrametric trees indicate the maximum likelihood transition point of the switch in branching rates, as estimated by a general mixed Yule-coalescent (GMYC) model. The GMYC analysis was performed using a single threshold. (**b**) Lineages-through-time plot based on the time calibrated tree obtained with all 176 COI unique haplotypes. The sharp increase in branching rate, corresponding to the transition from interspecies to intra-species branching events, is indicated by a vertical red line. 95% confidence intervals estimated ranges from 116 to 118.

**Figure 3:**
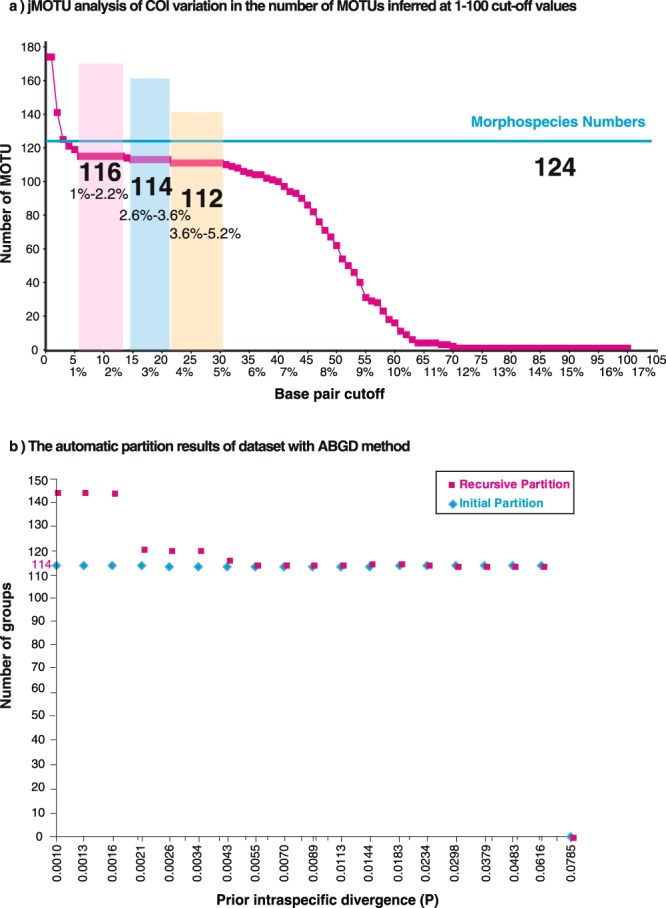
Species delimitations based on distance threshold. (**a**) jMOTU analysis of COI variation in the number of MOTUs inferred at 1–100 cut-off values. Critical cutoff intervals are indicated with shaded sections. (**b**) Automatic partition of the COI gene from Automatic Barcode Gap Discovery (ABGD).

The single-threshold GMYC analysis of the 176 unique haplotypes (Fig. 2) suggested that the likelihood of the GMYC model (1039.17) was significantly higher than that of the null model of uniform (coalescent) branching rates (943.58), with a likelihood ratio 191.18. GMYC inferred 117 species, with a confidence interval ranging from 116 to 118 at the 95% confidence level (Fig. 2). The BIN (Barcode Index Number)-RESL (Refined single linkage algorithm, which clusters the sequences in BOLD into BINs) system implemented in BOLD performed very similarly to GMYC, finding 114 BINs/MOTU (see Supplementary Table S1). jMOTU revealed three plateaus of MOTU richness over three ranges of percentage sequence divergence cutoff values: 116 MOTU at cutoffs between 1–2.2% sequence divergences (equivalent to a 6–13 bp cutoff value), 114 MOTU at cutoffs between 2.6–3.6% sequence divergences (equivalent to a 15–21 bp cutoff value) and 112 MOTU at cutoffs between 3.6–5.2% sequence divergences (equivalent to a 22–30 bp cutoff value) (Fig. 3a). ABGD produced 114 groups (Fig. 3b), compatible with the jMOTU result at cutoffs between 2.6–3.6% sequence divergences.

Seven morphospecies of the genus *Hypena* (sp1, sp3, sp4, sp5, *H*. *squalida*, *H*. *rivuligera*, and *H*. *stygiana*) fell into one GMYC species (MOTU1); the morphospecies sp6 formed a separate GMYC species (MOTU2), the morphospecies sp2 and sp7 formed a single GMYC species (MOTU3), and *H*. *tristalis* and *H*. *kengkalis* formed individual GMYC species (MOTU4 and MOTU5 respectively) (Fig. 1a).

### Step 2: Multigene species delimitation (*Hypena*)

The ML, MP and NJ trees showed that *H*. *tristalis* and *H*. *kengkalis* were monophyletic and these morphospecies could be delimited unambiguously in step 1 (see Supplementary Figs S2–S4). sp6 was distinct for mtDNA and 28S rDNA, but indistinct from the cluster formed by sp2 and sp7 for EF-1α and Wgl; a fifth cluster comprised sp1, sp3, sp4, sp5, *H*. *squalida*, *H*. *stygiana* and *H*. *rivuligera*. In the gene trees (ML, MP and NJ) of EF-1α, Wgl, and the tree of combined nDNA, one individual of *H*. *tristalis* fell within the lineage of sp1, sp3, sp4, sp5, *H*. *squalida*, *H*. *stygiana* and *H*. *rivuligera*. There were no readily apparent gaps between intraspecific and interspecific divergences in these datasets (see Supplementary Fig. S5 and Table S2).

All jMOTU analyses resulted in fewer than 12 MOTU; the number of morphospecies (Fig. 1b, Supplementary Fig. S6a). For COI and COII, there were plateaus of MOTUs richness at cutoffs between 0.6–5% and 0.53–3.68% divergences respectively, both yielding five MOTU. The nuclear genes (28S rDNA, EF-1α and Wgl) yielded fewer MOTU, showing plateaus with two, three and two MOTU at cutoffs between 0.64–1.15%, 0.84–2.85% and 0.5–4.5% divergences respectively. For three combined datasets (mtDNA, nDNA and mt + nDNA), there were plateaus at cutoffs between 0.5–4.5%, 0.8–1.7% and 0.37–1.14% (1.14–2.1%) sequence divergence, yielding five, three and five (six) MOTU respectively (Fig. 1b, Supplementary Fig. S6b). In the ABGD analysis (Fig. 1b, Supplementary Fig. S7) almost all results indicated that initial partitions were consistent with recursive partitions, with the number of MOTU ranging from 1 to a large number (corresponding to MOTU of unique sequences). The initial partition divided the 12 morphospecies into 5, 5, 2, 3, 2, 5, 3 and 5 MOTU for COI, COII, 28S rDNA, EF-1α, Wgl, mtDNA, nDNA and mt + nDNA respectively, consistent with the results of the jMOTU analysis.

The likelihood ratios of the GMYC model for COI, mtDNA and EF-1a were significantly higher than those of the null model of uniform (coalescent) branching rates while those for COII, 28S rDNA and Wgl were not (Fig. 1b, Table 1). COI, COII and combined mtDNA all inferred 5 GMYC species, with a confidence interval ranging from 5 to 6 (Table 1), while the 28S rDNA, EF-1α and Wgl gene estimated six (CI: 1–15), four (CI: 3–11) and six (CI: 2–12) GMYC species respectively (see Supplementary Fig. S8C–E), all less than the 12 morphospecies. Note that the *Hypena* specimens formed five BINs (based on COI), with an identical species makeup to the five MOTU found by the GMYC analysis.

**Table 1:** Lineage branching pattern fit to single threshold variants of the GMYC model.

Locus	No. of variable sites	No. of parsimony information sites	T	No. GMYC	CI	L_0_	L_GMYC_	L_R_
COI	93	77	0.005305	5	5–6	51.20422	56.3226	10.23677**
COII	52	45	0.004963	5	5–6	46.92273	48.79651	3.747559
28S	22	19	0.001335	6	1–15	104.8462	106.175	2.657676
EF-1a	42	37	0.003297	4	3–11	110.7959	113.8099	6.027846*
Wlg	34	25	0.002881	6	2–12	125.7286	127.7999	4.142599
mtDNA	287	229	0.005022	5	5–6	50.56622	55.3321	8.45875*

T, threshold genetic distance from the branch tips where transition occurred (presented for single-threshold models). No. GMYC, number of putative species as the sum of sequence clusters and singletons. CI, confidence intervals as solutions within 2 log-likelihood units of the maximum likelihood. L_0_, likelihood for null model. L_GMYC_, likelihood for GMYC model. L_R_, significance of the likelihood ratio evaluated using a chi-square test with 3 degrees of freedom to compare GMYC and null models. **p < 0.01,*p < 0.05.

**Figure 4:**
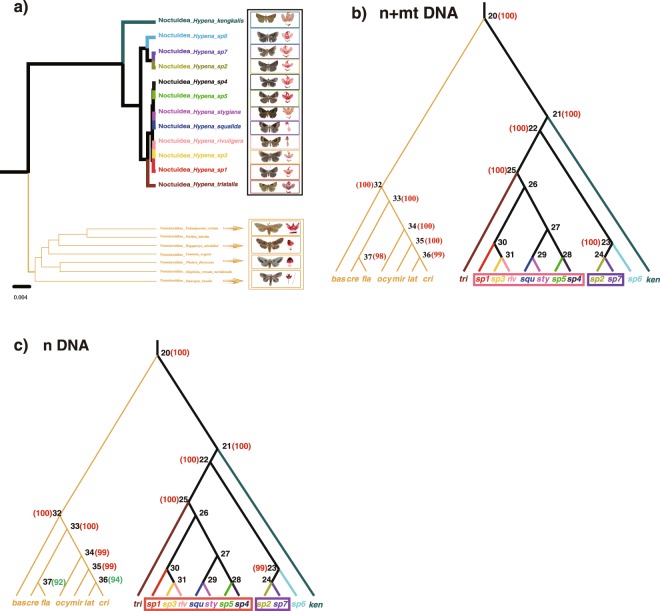
Species tree based upon combined analysis of five loci and posterior probabilities of each node for different datasets. (**a**) Species relationships among 12 *Hypena* species and 7 Notodontidae (outgroup) species. (**b**,**c**) Posterior probabilities of species delimitation with different datasets: 5 loci n + mtDNA (COI + COII + 28S rDNA + EF-1α + Wgl); 3loci nDNA (28S rDNA + EF-1α + Wgl) and 2 loci mtDNA (COI + COII); numbers in brackets beside node numbers indicate posterior probability support (red numbers are greater than or equal to 95, green numbers are less than 95, numbers less than 50 not shown).

The multigene species delimitation analysis (BPP) provided extremely weak support for the distinctiveness of the 12 morphospecies (Fig. 4; Supplementary Table S3). With rjMCMC, the posterior probability of the best delimitation model is 0.63, where sp2 and sp7 collapsed into one species and sp1, sp3, sp4, sp5, *H*. *squalida*, *H*. *stygiana* and *H*. *rivuligera* collapsed into another single species. Analysis of the nDNA data set gave similar results as the mt + nDNA data with a posterior tree probability of 0.4902. For data sets mt + nDNA and nDNA, the posterior probabilities for every speciation event also inferred the hypothesis that sp1, sp3, sp4, sp5, *H*. *squlida*, *H*. *stygiana* and *H*. *rivuligera* are one species and that sp2 and sp7 are another. All analyses confirm that sp6, *H*. *tristalis* and *H*. *kengkalis* are three distinct species.

### Geometric morphometrics

**Figure 5:**
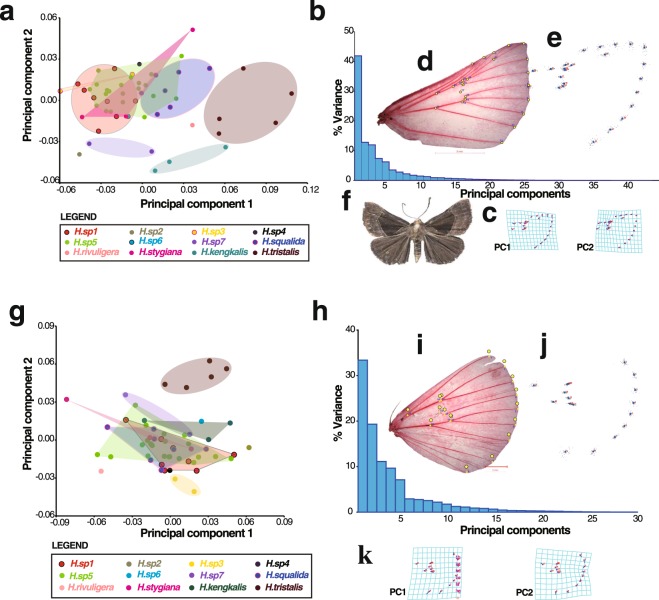
Geometrical morphology analysis of 55 *Hypena* specimens with forewings and hind wings respectively. (**a**,**g**) Scatter plots of the scores for the first two PCs (PC1 and PC2). (**b**,**h**) Percentages of total variance accounted for by PCs. (**c**,**k**) Transformation grids for visualizing shape change of forewing and hind wing (for PC1 and PC2). (**d**,**i**) Location of the wing landmarks used in the morphometric analysis, 24 and 17 landmarks for forewing and hind wing respectively. (**e**,**j**) Procrustes superimposition of the forewings and hindwings. (**f**) Dorsal view of *Hypena* (Schrank, 1802).

Geometric morphometric analyses found a strong correlation between the tangent shape and shape space (*R*^2^ = 0.99, *P* < 0.0001). The total variances of forewings and hindwings were 0.00314 and 0.00254 respectively, indicating that there was a small amount of shape variation in the sample. The PCA of the forewing and hindwing extracted 44 and 30 principal components with percentage of variation explained decreasing from 41.905% to 0.002% and 41.905% to 0.002% respectively (Fig. 5). The first two PCs accounted for 54.74% and 54.72% of the variation respectively (Fig. 5). The variation within and between species along the first two PC axes is shown in Fig. 5a,g. In the forewing analysis, the scatter plots showed that many of the taxa containing more than one specimen: sp1, *H*. *squalida*, sp7, *H*. *tristalis* and *H*. *kengkalis* divided into different subgroups and were independent of each other, but sp5, sp1, *H*. *squalida*, sp3 and *H*. *stygiana* overlapped one another to form a single large group. Two taxa comprising one individual each, sp6 and sp4, were also to be found in this group, although sp6 was distinct for mtDNA and 28S rDNA (see above). The two remaining taxa, with a single individual each, sp2 and *H*. *rivuligera* separated from other taxa. sp2 fell close to the sp7 cluster, which it group with in the DNA analysis (see above). The third, fourth and fifth PCs contributed 11.94%, 7.45%, and 6.25% of the total variance, respectively, which did not improve the separation of the overlapping morphospecies (Fig. 5a; Supplementary Fig. S9) (p > 0.05). The specimens formed more distinct clusters based on forewing shape than based on hindwing shape. Almost all taxa overlapped except for sp3, *tristalis*, *H*. *rivuliger*a and sp2, and of these sp2 and *H*. *rivuligera* were only represented by single specimens.

## Discussion

We used a two-step molecular approach to delimit species of moths from Dongling Mountain (Beijing, China). In the first step, standard species delimitation methods (GMYC, BIN-RESL, jMOTU, ABGD) were used with the standard, single-locus, animal DNA barcoding marker. These methods showed high congruence with the 124 morphospecies designations (117 GMYC species, 114 BINs; 116 jMOTU; 114 ABGD MOTU). Most (90%) of the conflicts were found in a single genus, *Hypena*. In the second step, restricted to 55 *Hypena* specimens representing 12 morphospecies, a multigene (COI, COII, 28S rDNA, EF-1α, Wgl) approach with analytical methods developed for multigene datasets was used. There were substantial differences between the morphospecies and molecular species limits, with only five molecularly distinct taxa. A geometric morphometric analysis was also used to delimit these specimens to operational taxonomic units, and was partly congruent with the molecular species delimitation (three species formed discrete groups, which were concordant with three of the five molecular groups).

Our methods included performing a species delimitation analysis with each gene fragment separately using the GMYC approach. GMYC is among the most commonly used species delimitation methods using single loci^32,44^. However, like most species delimitation methods, GMYC largely relies on a robust sequence alignment. This is not problematic for protein-coding regions, such as the standard COI barcode, but for non-coding regions (28S rDNA), for example, the phylogenetic signal contained in “indels” (gaps) is largely ignored. This could explain why 28S rDNA, assessed using GMYC, indicated a wider range of MOTU richness than the mtDNA. Other analyses that we applied to our datasets were BIN-RESL, jMOTU and ABGD. All three approaches using COI gave almost identical results to the use of GMYC. None of the analytical approaches fully resolved the twelve identified *Hypena* morphospecies.

The Bayesian approach BPP is a popular species delimitation method for multiple loci^33^, and uses a reversible jump Markov chain Monte Carlo approach to compute the posterior probability of the proposed nodes of the species tree; it performs better than some other general methods like spedeSTEM in simulations^45^. In BPP analysis, a species tree is needed as the guide tree; this may be difficult to infer^46^, especially for non-coding regions. Only 5 MOTUs were estimated for the *Hypena* moth group using a multigene BPP approach based on the combined datasets (n + mtDNA, nDNA). BPP’s failure to use information contained in the indels of 28S rDNA, together with the use of slowly evolving markers (EF-1α and Wgl), may have contributed to a similar estimate of MOTU richness as obtained with only COI despite using the additional character data.

In addition to the molecular analyses, we extracted information on forewing and hindwing shapes, and used PCA analysis to obtain preliminary taxonomic clusters. The forewing data showed more patterning that the hindwing data, generally forming clusters very similar to the molecular clusters. Geometric morphometrics did not provide evidence of statistically distinct entities matching the morphospecies designations.

Standard COI DNA barcoding is generally thought to find more biodiversity and increase species richness than traditional taxonomic approaches by uncovering undescribed and cryptic species^1–5,9,13^. However, in contrast to many previous studies, our case study found significant reductions in species richness compared with traditional taxonomy (step 1: 114–117 MOTU vs 124 morphospecies; step 2: 5 MOTU vs 12 morphospecies). A decrease or increase in species richness using DNA barcoding methods may be ascribed to one or more factors. Incomplete lineage sorting and hybridization among species can lead to non-monophyly on phylogenetic trees and incongruence in gene and species topologies. In Lepidoptera, hybridization can occur in closely related and parapatric taxa, and for example has been recorded between *Hyalophora cecropia* and *Hyalophora columbia* (Saturniidae)^47^, *Helicoverpa armigera* and *Helicoverpa assulta* (Noctuidae)^48,49^, and *Dendrolimus punctatus* and *Dendrolimus tabulaeformis* (Lasiocampidae)^50^. While incomplete lineage sorting and hybridization are two distinct processes, they can separately, or in combination, affect gene topologies and approaches have been proposed to distinguish them^51,52^. In our study, group membership of *Hypena* MOTU was highly congruent for mtDNA and nuclear markers, suggesting that there was little or no gene exchange between groups^11^. The only notable exception was a single sequence of *H*. *tristalis* that in analyses of EF-1α and Wgl sequences (but not 28S rDNA and mtDNA) did not cluster with its conspecifics and was placed outside the *H*. *tristalis* MOTU. This exception may be a remnant of a past hybridization and warrants exploration with additional data. There is also the strong possibility that some of the morphospecies are “over-splits”, which may be attributable to the difficulty of examinations of genital morphology.

For most insect communities, we suggest that the two step approach we used to delimit moth species in this case study is both cost and time-effective. The first step involves delimitation using the standard COI barcode and single-locus species delimitation approaches, such as GMYC, BIN or ABGD, and the second step reexamines those specimens which could not be unequivocally resolved in step one using multiple loci and analytical approaches. The standard DNA barcode can effectively and rapidly delimit most species, while additional molecular markers can be used to provide stronger conclusions for any closely related species groups that the first step uncovers. However, caution needs to be taken in choosing suitable analysis methods. We also note that it is highly desirable to integrate all genetic, morphological, ecological and behavioral information in reaching any definitive conclusions^5,11,46^.

## Methods

### Sampling, morphospecies delimitation

339 specimens of moths (Lepidoptera) were sampled from Dongling mountain (Beijing, China), (E: 115°29′48.2′′; N: 40°01′48.5′′). 12 additional specimens were sampled from three other locations around Beijing, China (Baihua mountain E: 115°33′27.4′′; N: 39°51′14.8′′ Wuling mountain N: 40°38.217′; E: 115°27.688′ Miaofeng mountain E: 116°03.347′; N: 39°58.907′). Rapid sorting into morphospecies, including assigning Linnaean names where applicable, was conducted by taxonomists (H.L.H and C.S.W) based on morphological characteristics and resulted in 124 morphospecies belonging to 10 families and 84 genera. The sample included 43 specimens identified as *Hypena* from Dongling mountain and 12 additional specimens identified as *Hypena* from other locations around Beijing. The 55 *Hypena* specimens were assigned to 12 morphospecies based on size, colour, forewing venation and male genitalia (additional details are provided in Appendix1). Eight sequences from six species of Trichoptera were downloaded from NCBI Genbank (JN200412, JX682405, HE614036, JQ548020, JQ548019, HQ978796, HQ978797, JX682406) as outgroups for the construction of phylogenetic trees. Eight specimens of Notodontidae were used as outgroups for the construction of phylogenetic trees for the *Hypena* and for Bayesian-based species delimitation.

### DNA extraction, amplification, and sequencing

DNA was extracted from two or three legs of each specimen (freshly preserved in 100% ethanol) using a BioMed (Beijing) DNeasy kit, and the barcode region of the mitochondrial COI gene was amplified and sequenced with standard primers^53^ (Table 2). For *Hypena* specimens, we also amplified (using primers listed in Table 2) and sequenced the mitochondrial gene, COII, and three nuclear genes, 28S rDNA, EF-1α and Wgl. For COI, 28S rDNA, EF-1α, and Wgl, reactions with a total volume of 30 μl were prepared using 3 μl of DNA template, 10.8 μl ddH_2_O, 15 μl of Mix (Taq DNA Polymerase (recombinant), 0.05 units μl^−1^; MgCl_2_, 4 mM; dNTPs (dATP, dCTP, dGTP, dTTP), 0.4 mM), and 0.6 μl of each primer (10 μM). For COII, PCR reactions were prepared using 1 μl of DNA template, 19.15 μl ddH_2_O, 3 μl of buffer (Mg^2+^ Free), 3 μl MgCl_2_ (25 mM), 2.5 μl of dNTPs (2.5 mM each), 0.6 μl of each primer (10 μM), and 0.15 μl of Taq polymerase (5 units μl^−1^). For COI and 28S rDNA, samples were initially denatured at 94 °C for 5 min followed by 30 cycles of amplification (denaturation at 94 °C for 30 s, annealing at 50 °C for 30 s, extension at 72 °C for 1 min) with a final extension at 72 °C for 5 min. COII used the same conditions except for annealing at 45 °C for 30 s. Conditions for the amplification of EF-1α and Wgl followed Braby and colleagues^54^.

**Table 2:** Primers used in this article of each marker.

Gene	Primer name (forward or reverse)	Sequence (5′-3′)	Reference
COI	LCO1490(fwd)	GGTCAACAAATCATAAAGATATTGG	Folmer *et al*. 1994
	HCO2198(rev)	TAAACTTCAGGGTGACCAAAAAATCA	Folmer *et al*. 1994
COII	C2-J3399(fwd)	TCTATCGGACAYCAATGATAYTG	This study
	TK-N3796(rev)	ACTATAAAATGGTTTAAGAG	This study
28S	Mo6 (fwd)	CCCCCTGAATTTAAGCATAT	Braby *et al*.^54^
	D3A-r (rev)	TCCGTGTTTCAAGACGGGTC	Braby *et al*.^54^
*Wingless*	LepWG1 (fwd)	GARTGYAARTGYCAYGGYATGTCTGG	Braby *et al*.^54^
	LepWG2 (rev)	ACTICGCRCACCARTGGAATGTRCA	Braby *et al*.^54^
EF-1a	EF44 (fwd)	GCYGARCGYGARCGTGGTATYAC	Braby *et al*.^54^
	EFxmf(fwd)	ACCTCCCAGGCTGATTGT	This study
	EFxmr(rev)	AACTCTTTGACGGACACG	This study
	EFrcM4 (rev)	ACAGCVACKGTYTGYCTCATRTC	Braby *et al*.^54^

Purified DNA fragments for each gene were sequenced with a range of forward (reverse for COII) primers (see Table 2) by BioMed (Beijing). Raw chromatograms were all checked manually by eye. After trimming the ends of the raw sequences, sequence alignment was performed using MUSCLE in MEGA7^55^ (default settings, −400 gap open, 1255 Max memory in MB, 8 Max iterations, 24 min diag length). Gaps in 28S rDNA sequences were treated as “Complete deletion”. All sequences were submitted to NCBI GenBank (see Supplementary Table S4 and Table S5).

### Species delimitation using COI barcodes

(i) GMYC analyses used the R package SPLITS (SPecies’ LImits by Threshold Statistics, version 2.10, https://r-forge.r-project.org/ projects/splits/)^32,44^, employing the single-threshold model which estimates the transition from coalescent to speciation branching patterns on an ultrametric tree. Analyses were completed on a reduced matrix that included only the 172 unique haplotypes. The selection of the most suitable model of DNA substitution (COI, HKY + I + G) was performed using ModelTest 3.7^56^ under the Bayesian Information Criterion^57^. BEAST v. 1.8.0^58^ was used to construct a maximum clade credibility summary tree with HKY + I + G and strict clock models on an arbitrary timescale. Analyses were run for 10 million generations, sampled every 50000 generations, with parameters estimated over the final 1000 generations. The output was diagnosed for convergence using Tracer v.1.4^59^, and summary statistics and trees were generated using the 10 million generations with TreeAnnotator v1.4.3^59^.
(ii) Sequences were submitted to BOLD as project “LEPDL”, MOTUs were generated from identified and unidentified sequences using the Refined Single Linkage algorithm (RESL). Sequences were assigned to MOTUs independent of the BIN (Barcode Index Numbers)^60^ registry.
(iii) jMOTU v1.0.6^30^, a Java program for the analysis of DNA barcode datasets based on an explicit threshold, was used to cluster sequences into groups that differed by fixed pairwise distances. Analyses were repeated at cut-off values of 1–100 bp after pre-experiments. 95% values were set for the minimum overlap required between sequences and 97% values for the low megablast identity filter parameter; MOTUs were then inferred from cutoff values.
(iv) The COI dataset was submitted to the ABGD website (http://wwwabi.snv.jussieu.fr/public/abgd/), using the default gap width X = 1.5 and setting prior intraspecific divergences from P = 0.001 to P = 0.1 with 20 steps and K2P distances; other parameters used default conditions.

### Species delimitation for *Hypena* using additional molecular markers

#### Species delimitation using additional markers

Firstly, Maximum Likelihood (ML), Neighbor-Joining (NJ) and Maximum Parsimony (MP) methods were used to construct trees for the 55 *Hypena* specimens (additional details about tree building are provided in Appendix 2), and gene regions were analysed both separately (COI, COII, 28S rDNA, EF-1α and Wgl) and in combinations (mt + nDNA, nDNA, and mtDNA). Secondly, the five loci (COI, COII, 28S rDNA, EF-1α and Wgl) were analyzed individually with GMYC, BINs, jMOTU, and ABGD methods as described above. We further explored the intraspecific and interspecific variations within this closely related species group and calculated K2P sequence divergences for each gene and for the combinations using a perl script developed for this task^61,62^.

#### Multigene species delimitation - BPP analysis

We used multigene sequence data to delimit species with Bayesian software BPP v2.1^33^. We generated a guide species tree using *BEAST^63^ using best-fit nucleotide substitution models (28S rDNA, COII, mt + nDNA, nDNA, and mtDNA, GTR + I + G; COI, GTR + G; EF-1α, TN93 + I + G; Wgl, TN93 + G) and strict clocks for all five loci. Analyses were run for 5 million generations (sampled every 50,000 generations, with parameters estimated over the final 1,000 generations) following a Yule process and a constant population size model.

This guide species tree was then entered into BPP v2.1^33^, with equal prior probabilities given to each alternative rooted species tree compatible with it. We assigned the prior τ0 ∼ G (2, 20000), with mean 0.0001, and θ ∼ G (2, 2000), with mean 0.001. For this step, we used two methods, these being with and without rjMCMC (Reversible-Jump Markov Chain Monte Carlo), and for each method we used two datasets for species delimitation - (i) full (all 5 loci) and (ii) nDNA only (3 loci). For the former method, that using rjMCMC, the two alternative rjMCMC algorithms and different fine-tune parameters gave the same results; final analyses were conducted using algorithm 0 with fine-tuning parameter e = 20; the posterior probability P is the probability of forming two distinct species. The second method, referred to as the “τ threshold” method for species delimitation, does not require the use of rjMCMC. This approach involves integrating over only the most complex model (the fully resolved guide tree) using constant dimensional MCMC, and using the posterior distribution of species divergence times to identify the species delimitations. The posterior probability P, that the divergence time between a pair of putative species is below a threshold value (determined by the species definition), is interpreted as the probability that the two groups form a single species, whereas 1 − P is the probability that they form two distinct species. Each method was run twice to confirm consistency. All analyses were run for 100,000 generations (sampling interval = 5) with a burn-in of 20,000 generations. Trees generated prior to stationarity were discarded as burn-in^64^, and results were summarized with a majority-rule consensus tree from the remaining trees from the four independent runs. Bayesian posterior probabilities (PP) were assessed at all nodes and clades with PP ≥0.95 were considered strongly supported^65^.

### Morphometric delimitation of *Hypena*

For each *Hypena* specimen, right or left forewings and hindwings were dissected and mounted using standard techniques^66,67^. Slides were photographed and images imported into tps-UTILS 1.43^68^ to create tps files. 24 forewing and 17 hindwing homologous landmarks were positioned on wing venation nodes. Cartesian coordinates of landmarks were digitized with tps-DIG 1.43^69^. The effect of measurement error was assessed for 55 forewings that had been digitized twice through a Procrustes ANOVA of shape^70^. The measurement error was 0.0181% of the total variation for shape variables, and 0.0007% for centroid size. tps-Small^71^ was used to test whether observed variation in shape was sufficiently small that the distribution of points in tangent space gave a good approximation of their distribution in shape space. MorphoJ^72^ was used for principal component analyses (PCA).

## Electronic supplementary material

Supplementary Figs

Supplementary tables

Appendix1–2
