# Supplementary material for: A two-step DNA barcoding approach for delimiting moth species: moths of Dongling Mountain (Beijing, China) as a case study
Source: Sci Rep. 2018 Sep 24;8:14256. doi: 10.1038/s41598-018-32123-9 (PMC6155206; doi:10.1038/s41598-018-32123-9)
Supplement: Supplementary file 1 — Supplementary Figs [file 41598_2018_32123_MOESM1_ESM.pdf]

A two-step DNA barcoding approach for delimiting moth species: moths of Dongling Mountain (Beijing, China) as a case study

Qian Jin[1†], Xi-Min Hu[2†], Hui-Lin Han[3], Fen Chen[1], Wei-Jia Cai[1], Qian-Qian Ruan[1], Bo Liu[1], Gui-Jie Luo[1], Hao Wang[1], Xu Liu[1], Robert D. Ward[4], Chun-Sheng Wu[5], John-James Wilson[6,7] and Ai-Bing Zhang[2*]

[1]*Suqian Institute of Agricultural Sciences, Jiangsu Academy of Agricultural Sciences, Suqian, Jiangsu 223800*

[2]*College of Life Sciences, Capital Normal University, Beijing 100048, China*

[3]*School of Forestry, Experiment Center, Northeast Forestry University, Haerbin 150040, China*

[4]*CSIRO National Research Collections Australia, GPO Box 1538, Hobart, Tasmania 7001, Australia*

[5]*Institute of Zoology, Chinese Academy of Sciences, 1 Beichen West Road, Chaoyang District, Beijing 100101, China*

[6]*School of Applied Sciences, Faculty of Computing, Engineering and Science, University of South Wales, Pontypridd, CF37 4AT, United Kingdom*

[7]*Department of Microbiology and Parasitology, Faculty of Medical Science, Naresuan University, Phitsanulok 65000, Thailand*

*Corresponding: E-mail: zhangab2008@gmail.com/zhangab2008@mail.cnu.edu.cn

†these authors contributed equally to this work

Supporting Information

FigureS1. Phylogenetic tree of Lepidoptera, inferred using neighbor-joining analysis of 351 COI sequences. Red colour highlights non-monophyletic at species level and blue colour highlights non-monophyletic at genus level.

FigureS2. Phylogenetic trees (ML) of 12 *Hypena* species with outgroups, constructed with different genes COI, COII, 28S, EF-1α,and Wgl, and in combination, mtDNA (COI, COII), nDNA(28S, EF-1α, Wgl) and mt+nDNA (COI, COII, 28S, EF-1α, Wgl).Clades with different colors indicate different species. Numbers above or below branches indicate bootstrap values(less than 50 not shown).

FigureS3. Phylogenetic trees (NJ) of 12 *Hypena* species with outgroups, constructed with different genes COI, COII, 28S, EF-1α, and Wgl, and in combination, mtDNA (COI, COII), nDNA(28S, EF-1α, Wgl) and mt+nDNA (COI, COII, 28S, EF-1α, Wgl). Clades with different colors indicate different species. Numbers above or below branches indicate bootstrap values(less than 50 not shown).

FigureS4. Phylogenetic trees (MP) of 12 *Hypena* species with outgroups constructed with different genes COI, COII, 28S, EF-1α, and Wgl,and in combination, mtDNA (COI, COII), nDNA(28S, EF-1α, Wgl) and mt+nDNA (COI, COII, 28S, EF-1α, Wgl). Clades with different colors indicate different species. Numbers above or below branches indicate bootstrap values (less than 50 not shown).

FigureS5. Frequency distribution of intraspecific and interspecific genetic divergence in *Hypena* with different data sets COI, COII, 28S, EF-1α, and Wgl, and in combination, mtDNA (COI, COII), nDNA(28S, EF-1α, Wgl) and mt+nDNA (COI, COII, 28S, EF-1α, Wgl). Total numbers of comparisons: 277 intraspecific and 1208 interspecific pairs across 12 *Hypena* species. Divergences calculated using Kimura's two parameter (K2P) model.

FigureS6. MOTU inferred in five loci and three combined gene categories of Hypena species using jMOTU method. a) Variation in the number of MOTUs inferred at different cut-off values (in the range 1-50 bp for COI, COII and combined gene nDNA, 1-30bp for 28S, EF-1α and Wgl genes,

and 1–120 bp for combined genes mtDNA and mt+nDNA). Critical cutoff intervals for the different datasets are indicated with shaded red sections. b) Comparison of morphological species identified with MOTU compositions at the critical cutoff intervals. Different colors represent different MOTUs.

FigureS7. Automatic partition of the eight single and combined locus data sets using ABGD (Automatic Barcode Gap Discovery) method.

FigureS8. GMYC analysis of 12 *Hypena* species with five single loci COI, COII, 28S, EF-1α, andWgl. Ultrametric trees of *Hypena* species constructed with different loci (left) and lineage-through-time plots (right). The red vertical lines on the ultrametric trees indicate the maximum likelihood transition points of the switch in branching rates, as estimated by a general mixed Yule-coalescent (GMYC) model. The GMYC analysis was performed using a single threshold.

FigureS9. Scatterplot of a principal component analysis (PCA) in shape space of forwings. Different colour represents different species which are largely overlapping along PC1 and PC2 in Fig 5. *Red - sp1,yellow - sp3,green - sp5,blue - H. squalida, fuschia - H. stygiana*.

FigureS1

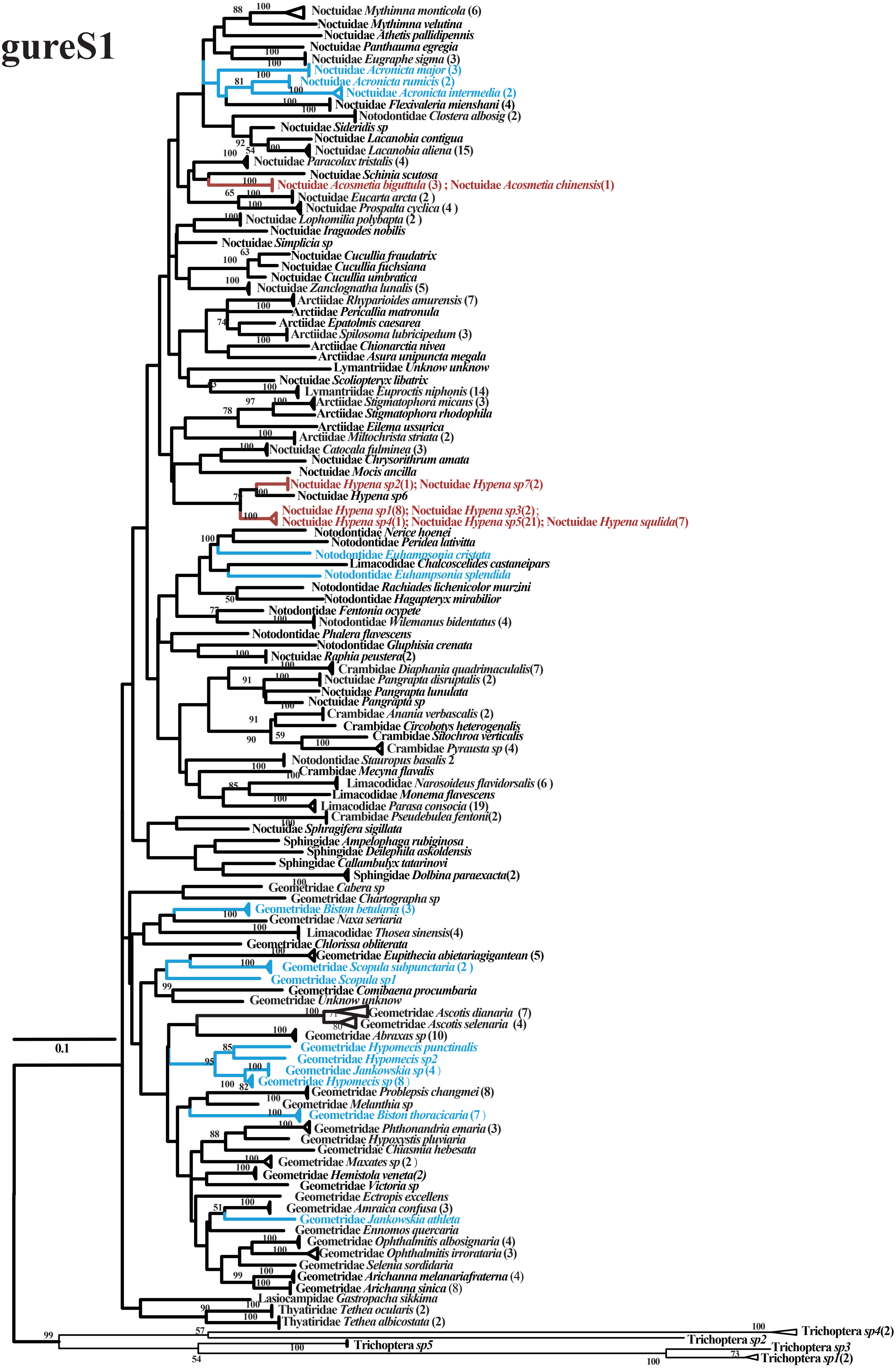

Noctuidae Mythimna monticola (6)
Noctuidae Mythimna velutina
Noctuidae Athetis pallidipennis
Noctuidae Panthauma egregia
Noctuidae Eugraphe sigma (3)
Noctuidae Acronicta major (3)
Noctuidae Acronicta rumicis (2)
Noctuidae Acronicta intermedia (2)
Noctuidae Flexivaleria mienshani (4)
Notodontidae Clostera albosig (2)
Noctuidae Sideridis sp
Noctuidae Lacanobia contigua
Noctuidae Lacanobia aliena (15)
Noctuidae Paracolax tristalis (4)
Noctuidae Schinia scutosa
Noctuidae Acosmetia biguttula (3) ; Noctuidae Acosmetia chinensis(1)
Noctuidae Eucarta arcta (2)
Noctuidae Prospalta cyclica (4)
Noctuidae Lophomilia polybapta (2)
Noctuidae Iragaodes nobilis
Noctuidae Simplicia sp
Noctuidae Cucullia fraudatrix
Noctuidae Cucullia fuchsiana
Noctuidae Cucullia umbratica
Noctuidae Zanclognatha lunalis (5)
Arctiidae Rhyparioides amurensis (7)
Arctiidae Pericallia matronula
Arctiidae Epatolmis caesarea
Arctiidae Spilosoma lubricipedum (3)
Arctiidae Chionarctia nivea
Arctiidae Asura unipuncta megala
Lymantriidae Unknow unknow
Noctuidae Scoliopteryx libatrix
Lymantriidae Euproctis niphonis (14)
Arctiidae Stigmatophora micans (3)
Arctiidae Stigmatophora rhodophila
Arctiidae Eilema ussurica
Arctiidae Miltochrista striata (2)
Noctuidae Catocala fulminea (3)
Noctuidae Chrysorithrum amata
Noctuidae Mocis ancilla
Noctuidae Hypena sp2(1); Noctuidae Hypena sp7(2)
Noctuidae Hypena sp6
Noctuidae Hypena sp1(8); Noctuidae Hypena sp3(2);
Noctuidae Hypena sp4(1); Noctuidae Hypena sp5(21); Noctuidae Hypena squlida(7)
Notodontidae Nerice hoenei
Notodontidae Peridea lativitta
Notodontidae Euhampsonia cristata
Limacodidae Chalcoscelides castaneipars
Notodontidae Euhampsonia splendida
Notodontidae Rachiades lichenicolor murzini
Notodontidae Hagapteryx mirabilior
Notodontidae Fentonia ocypete
Notodontidae Wilemanus bidentatus (4)
Notodontidae Phalera flavescens
Notodontidae Gluphisia crenata
Noctuidae Raphia peustera(2)
Crambidae Diaphania quadrimaculalis(7)
Noctuidae Pangrapta disruptalis (2)
Noctuidae Pangrapta lunulata
Noctuidae Pangrapta sp
Crambidae Anania verbascalis (2)
Crambidae Circobotys heterogenalis
Crambidae Sitochroa verticalis
Crambidae Pyrausta sp (4)
Notodontidae Stauropus basalis 2
Crambidae Mecyna flavalis
Limacodidae Narosoideus flavidorsalis (6)
Limacodidae Monema flavescens
Limacodidae Parasa consocia (19)
Crambidae Pseudebulea fentoni(2)
Noctuidae Sphragifera sigillata
Sphingidae Ampelophaga rubiginosa
Sphingidae Deilephila askoldensis
Sphingidae Callambulyx tatarinovi
Sphingidae Dolbina paraexacta(2)
Geometridae Cabera sp
Geometridae Chartographa sp
Geometridae Biston betularia (3)
Geometridae Naxa seriaria
Limacodidae Thosea sinensis(4)
Geometridae Chlorissa obliterata
Geometridae Eupithecia abietariagigantean (5)
Geometridae Scopula subpunctaria (2)
Geometridae Scopula sp1
Geometridae Comibaena procumbaria
Geometridae Unknow unknow
Geometridae Ascotis dianaria (7)
Geometridae Ascotis selenaria (4)
Geometridae Abraxas sp (10)
Geometridae Hypomecis punctinalis
Geometridae Hypomecis sp2
Geometridae Jankowskia sp (4)
Geometridae Hypomecis sp (8)
Geometridae Problepsis changmei (8)
Geometridae Melanthia sp
Geometridae Biston thoracicaria (7)
Geometridae Phthonandria emaria (3)
Geometridae Hypoxystis pluviaria
Geometridae Chiasmia hebesata
Geometridae Maxates sp (2)
Geometridae Hemistola veneta(2)
Geometridae Victoria sp
Geometridae Ectropis excellens
Geometridae Amraica confusa (3)
Geometridae Jankowskia athleta
Geometridae Ennomos quercaria
Geometridae Ophthalmitis albosignaria (4)
Geometridae Ophthalmitis irrorataria (3)
Geometridae Selenia sordidaria
Geometridae Arichanna melanariafraterna (4)
Geometridae Arichanna sinica (8)
Lasiocampidae Gastropacha sikkima
Thyatiridae Tethea ocularis (2)
Thyatiridae Tethea albicostata (2)
Trichoptera sp4(2)
Trichoptera sp2
Trichoptera sp5
Trichoptera sp3
Trichoptera sp1(2)
0.1

FigureS2

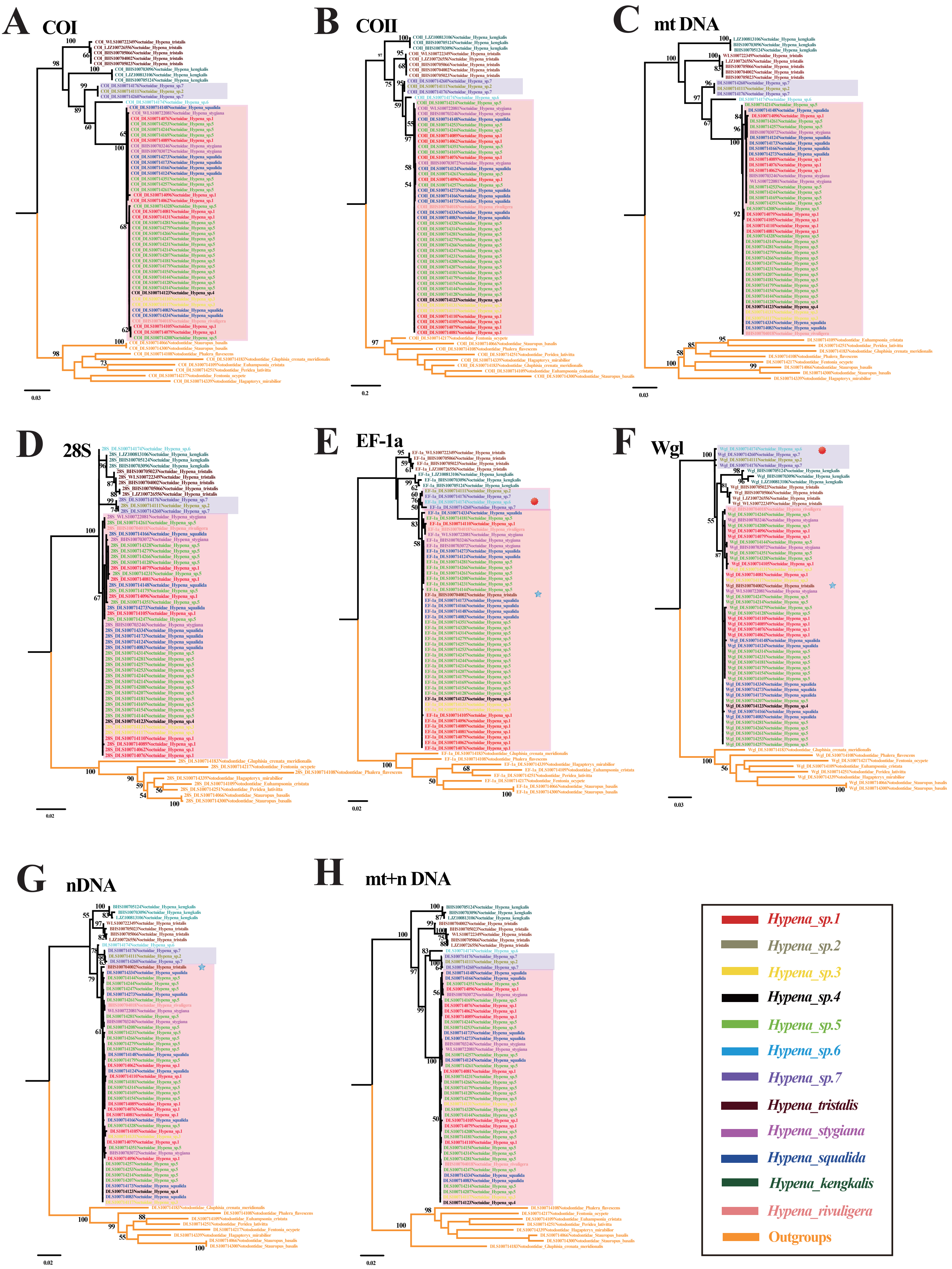
A COI
B COII
C mt DNA
D 28S
E EF-1a
F Wgl
G nDNA
H mt+n DNA
Hypena_sp.1
Hypena_sp.2
Hypena_sp.3
Hypena_sp.4
Hypena_sp.5
Hypena_sp.6
Hypena_sp.7
Hypena_tristalis
Hypena_stygiana
Hypena_squalida
Hypena_kengkalis
Hypena_rivuligera
Outgroups

FigureS3

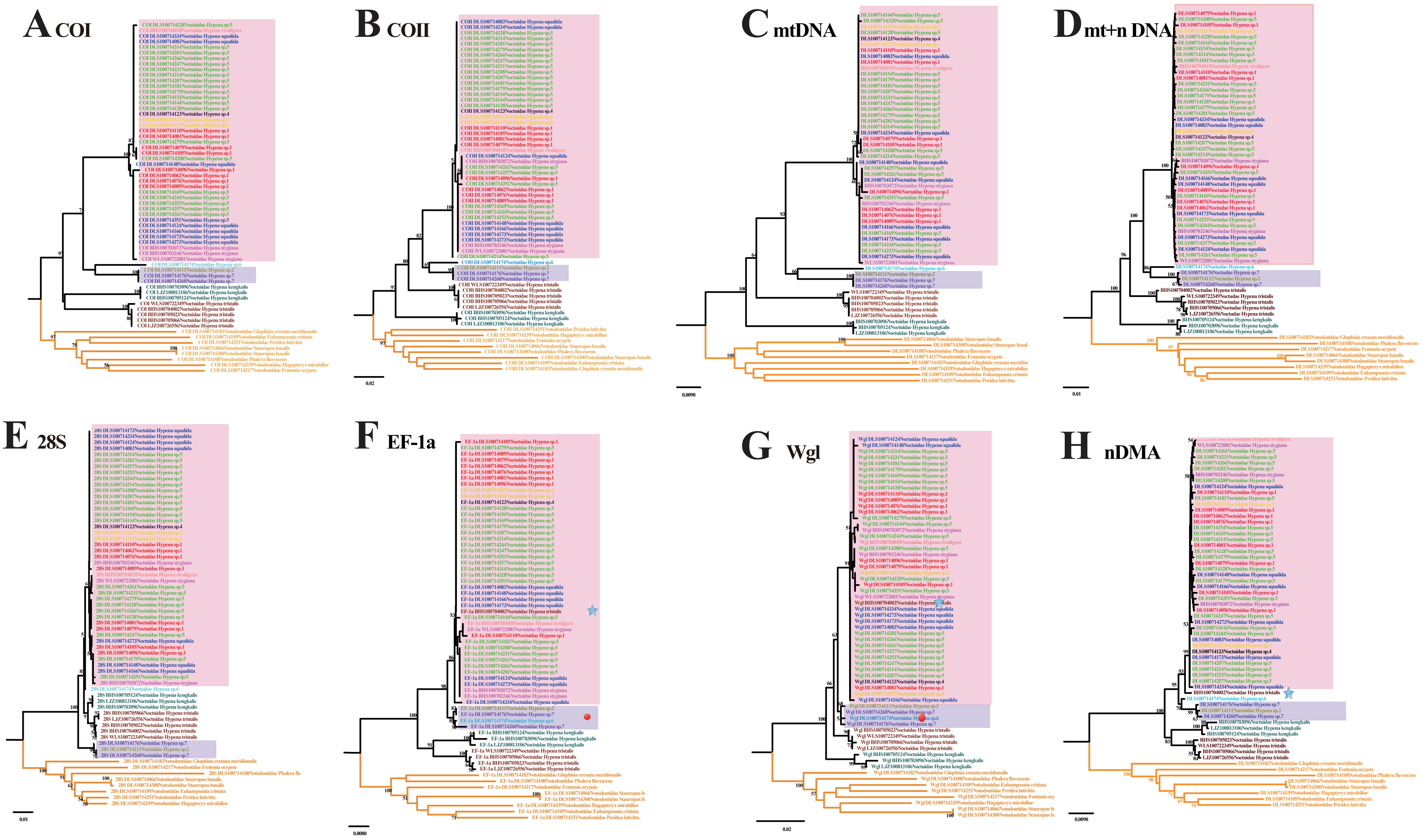

FigureS4

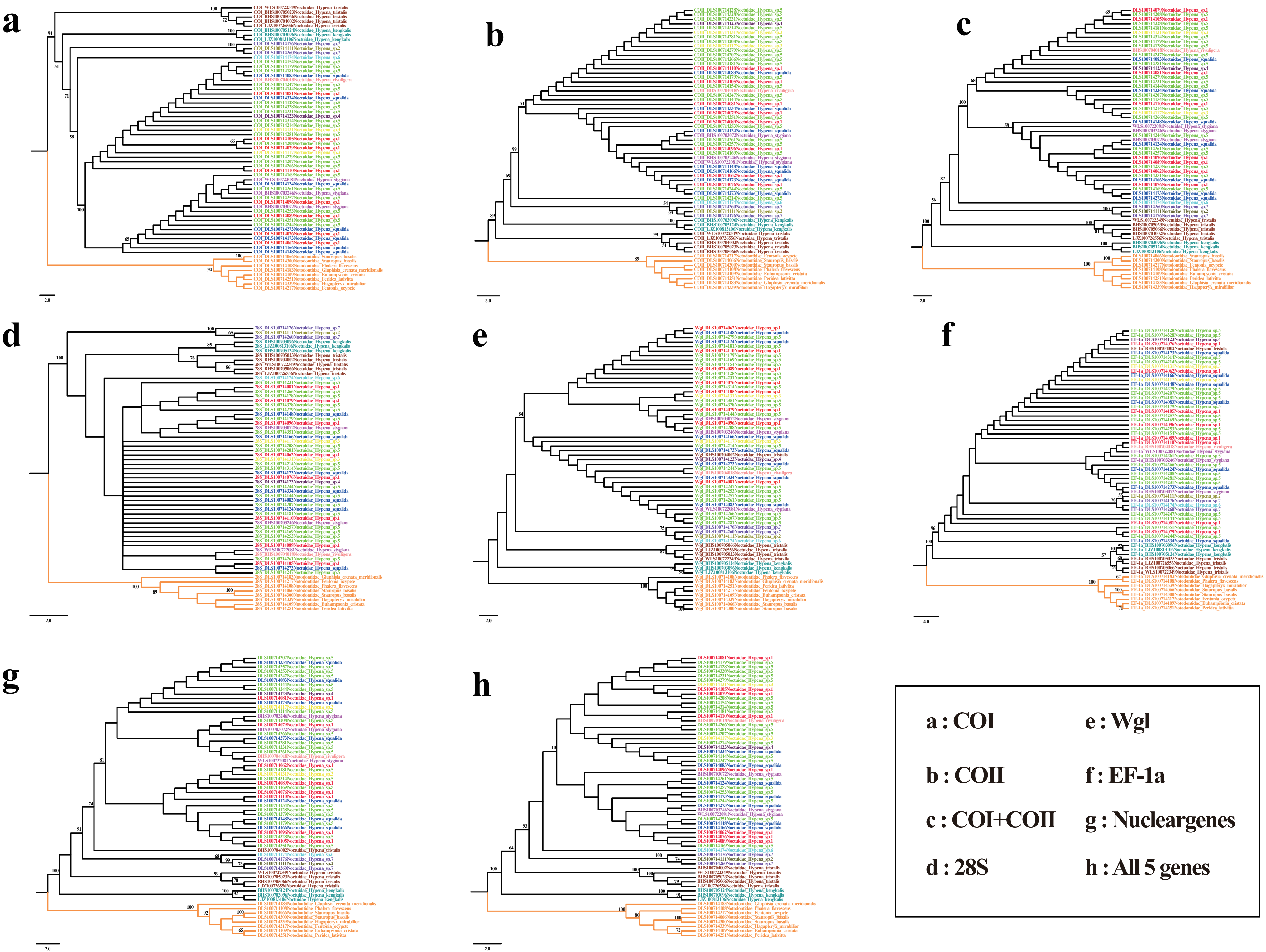
a
b
c
d
e
f
g
h
a : COI
e : Wgl
b : COII
f : EF-1a
c : COI+COII
g : Nucleargenes
d : 28S
h : All 5 genes

FigureS5

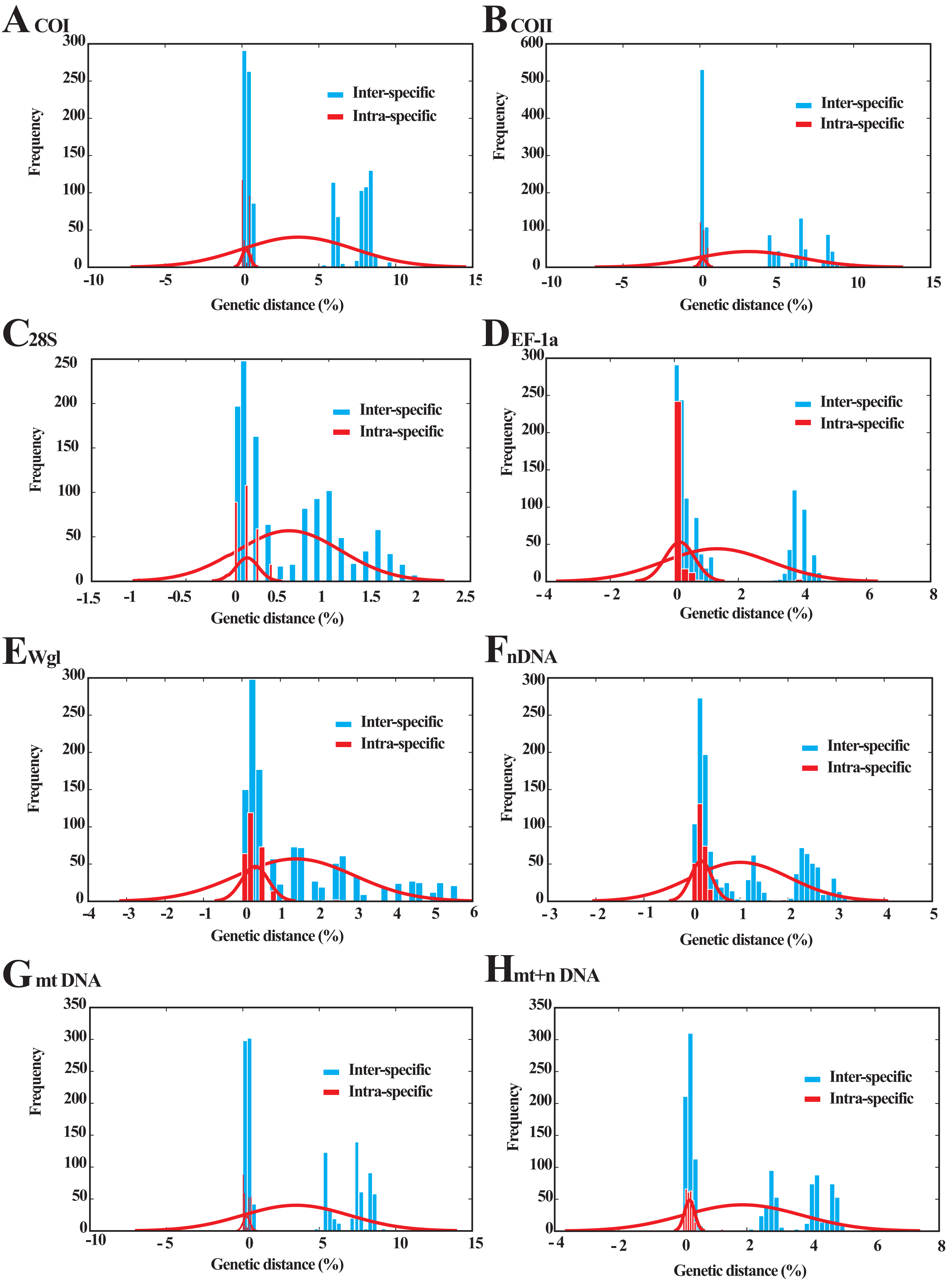
A COI
Frequency
Genetic distance (%)
Inter-specific
Intra-specific
B COII
Frequency
Genetic distance (%)
Inter-specific
Intra-specific
C 28S
Frequency
Genetic distance (%)
Inter-specific
Intra-specific
D EF-1a
Frequency
Genetic distance (%)
Inter-specific
Intra-specific
E Wgl
Frequency
Genetic distance (%)
Inter-specific
Intra-specific
F nDNA
Frequency
Genetic distance (%)
Inter-specific
Intra-specific
G mt DNA
Frequency
Genetic distance (%)
Inter-specific
Intra-specific
H mt+n DNA
Frequency
Genetic distance (%)
Inter-specific
Intra-specific

FigureS6

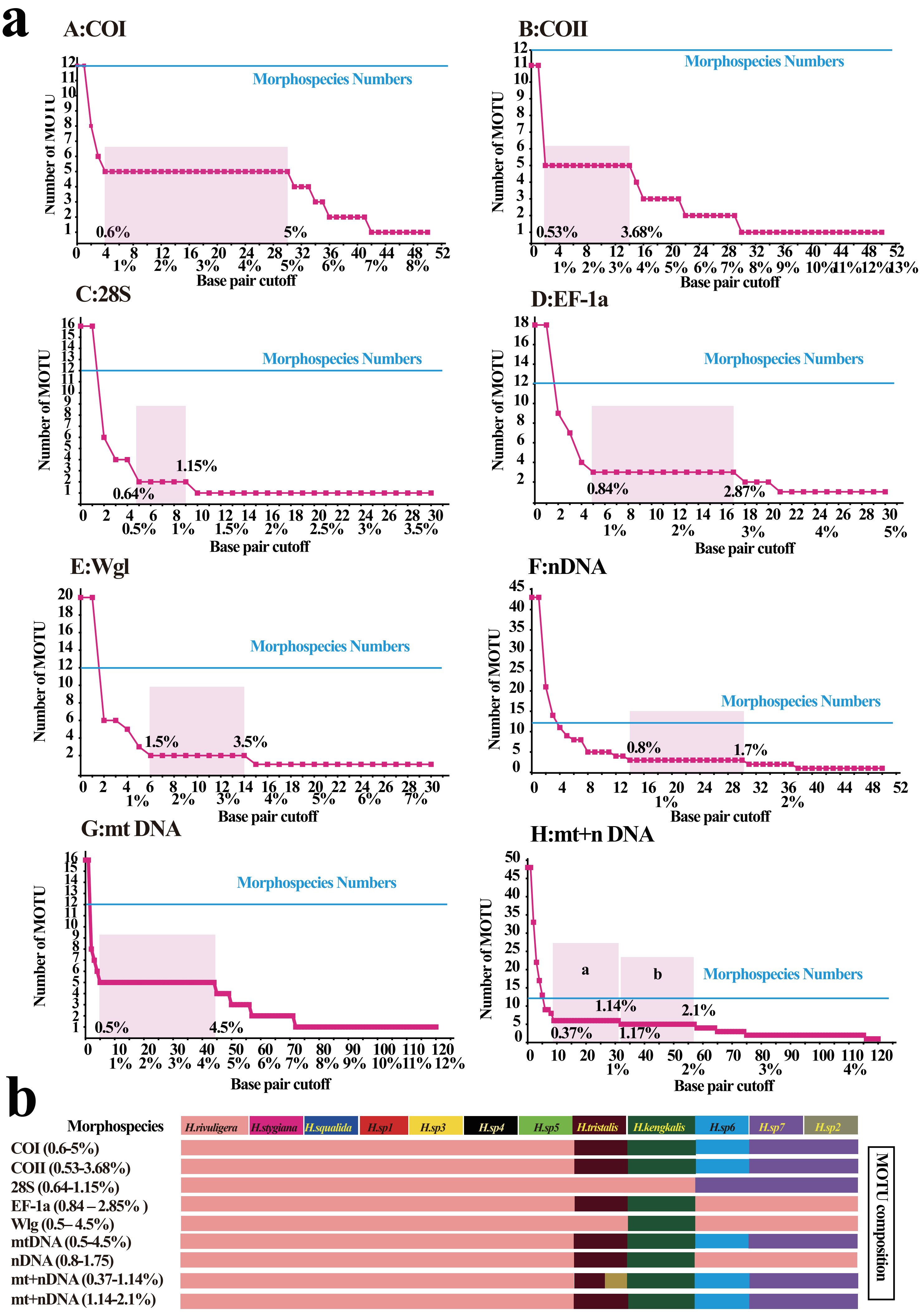

a
A:COI
Morphospecies Numbers
Number of MOTU
0.6%
5%
Base pair cutoff
B:COII
Morphospecies Numbers
Number of MOTU
0.53%
3.68%
Base pair cutoff
C:28S
Morphospecies Numbers
Number of MOTU
1.15%
0.64%
Base pair cutoff
D:EF-1a
Morphospecies Numbers
Number of MOTU
0.84%
2.87%
Base pair cutoff
E:Wgl
Morphospecies Numbers
Number of MOTU
1.5%
3.5%
Base pair cutoff
F:nDNA
Morphospecies Numbers
Number of MOTU
0.8%
1.7%
Base pair cutoff
G:mt DNA
Morphospecies Numbers
Number of MOTU
0.5%
4.5%
Base pair cutoff
H:mt+n DNA
Morphospecies Numbers
Number of MOTU
a
b
1.14%
2.1%
0.37%
1.17%
Base pair cutoff
b
Morphospecies
H.rivuligera
H.stygiana
H.squalida
H.sp1
H.sp3
H.sp4
H.sp5
H.tristalis
H.kengkalis
H.sp6
H.sp7
H.sp2
COI (0.6-5%)
COII (0.53-3.68%)
28S (0.64-1.15%)
EF-1a (0.84 – 2.85%)
Wlg (0.5– 4.5%)
mtDNA (0.5-4.5%)
nDNA (0.8-1.75)
mt+nDNA (0.37-1.14%)
mt+nDNA (1.14-2.1%)
MOTU composition

FigureS7

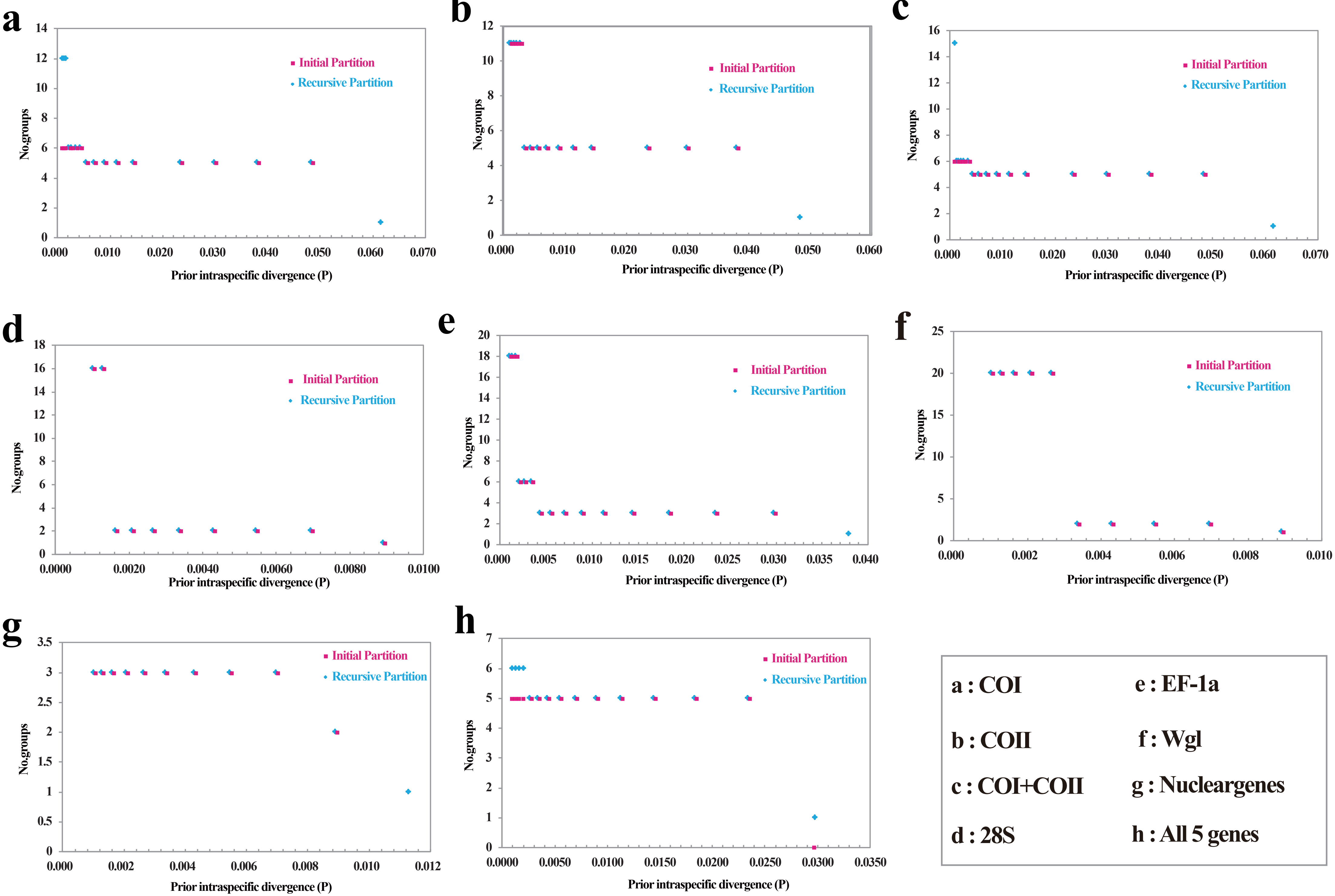
a
No.groups
Prior intraspecific divergence (P)
Initial Partition
Recursive Partition
b
No.groups
Prior intraspecific divergence (P)
Initial Partition
Recursive Partition
c
No.groups
Prior intraspecific divergence (P)
Initial Partition
Recursive Partition
d
No.groups
Prior intraspecific divergence (P)
Initial Partition
Recursive Partition
e
No.groups
Prior intraspecific divergence (P)
Initial Partition
Recursive Partition
f
No.groups
Prior intraspecific divergence (P)
Initial Partition
Recursive Partition
g
No.groups
Prior intraspecific divergence (P)
Initial Partition
Recursive Partition
h
No.groups
Prior intraspecific divergence (P)
Initial Partition
Recursive Partition
a : COI
e : EF-1a
b : COII
f : Wgl
c : COI+COII
g : Nucleargenes
d : 28S
h : All 5 genes

FigureS8

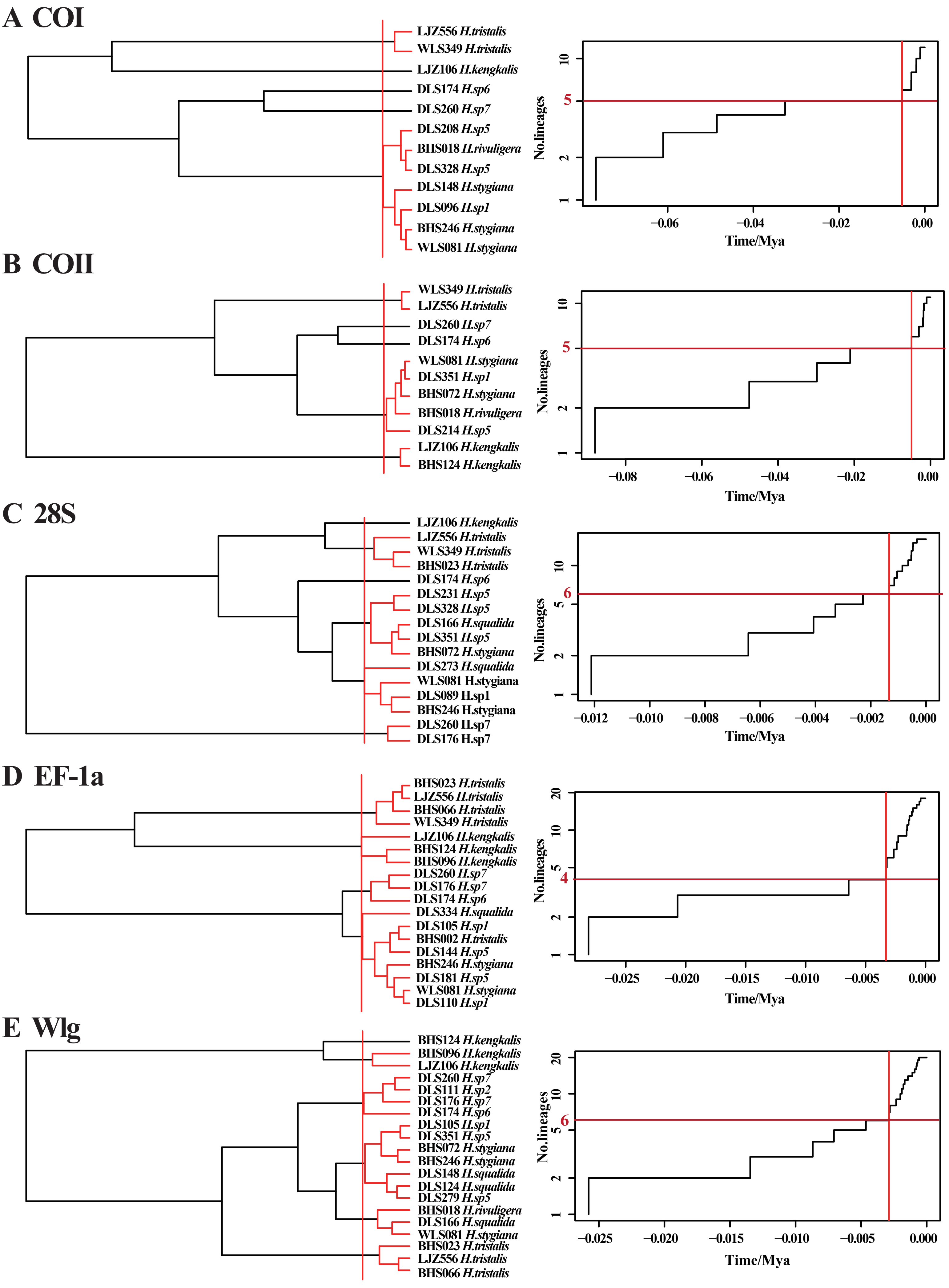
A COI
LJZ556 H.tristalis
WLS349 H.tristalis
LJZ106 H.kengkalis
DLS174 H.sp6
DLS260 H.sp7
DLS208 H.sp5
BHS018 H.rivuligera
DLS328 H.sp5
DLS148 H.stygiana
DLS096 H.sp1
BHS246 H.stygiana
WLS081 H.stygiana
No.lineages
Time/Mya
B COII
WLS349 H.tristalis
LJZ556 H.tristalis
DLS260 H.sp7
DLS174 H.sp6
WLS081 H.stygiana
DLS351 H.sp1
BHS072 H.stygiana
BHS018 H.rivuligera
DLS214 H.sp5
LJZ106 H.kengkalis
BHS124 H.kengkalis
No.lineages
Time/Mya
C 28S
LJZ106 H.kengkalis
LJZ556 H.tristalis
WLS349 H.tristalis
BHS023 H.tristalis
DLS174 H.sp6
DLS231 H.sp5
DLS328 H.sp5
DLS166 H.squalida
DLS351 H.sp5
BHS072 H.stygiana
DLS273 H.squalida
WLS081 H.stygiana
DLS089 H.sp1
BHS246 H.stygiana
DLS260 H.sp7
DLS176 H.sp7
No.lineages
Time/Mya
D EF-1a
BHS023 H.tristalis
LJZ556 H.tristalis
BHS066 H.tristalis
WLS349 H.tristalis
LJZ106 H.kengkalis
BHS124 H.kengkalis
BHS096 H.kengkalis
DLS260 H.sp7
DLS176 H.sp7
DLS174 H.sp6
DLS334 H.squalida
DLS105 H.sp1
BHS002 H.tristalis
DLS144 H.sp5
BHS246 H.stygiana
DLS181 H.sp5
WLS081 H.stygiana
DLS110 H.sp1
No.lineages
Time/Mya
E Wlg
BHS124 H.kengkalis
BHS096 H.kengkalis
LJZ106 H.kengkalis
DLS260 H.sp7
DLS111 H.sp2
DLS176 H.sp7
DLS174 H.sp6
DLS105 H.sp1
DLS351 H.sp5
BHS072 H.stygiana
BHS246 H.stygiana
DLS148 H.squalida
DLS124 H.squalida
DLS279 H.sp5
BHS018 H.rivuligera
DLS166 H.squalida
WLS081 H.stygiana
BHS023 H.tristalis
LJZ556 H.tristalis
BHS066 H.tristalis
No.lineages
Time/Mya

FigureS9

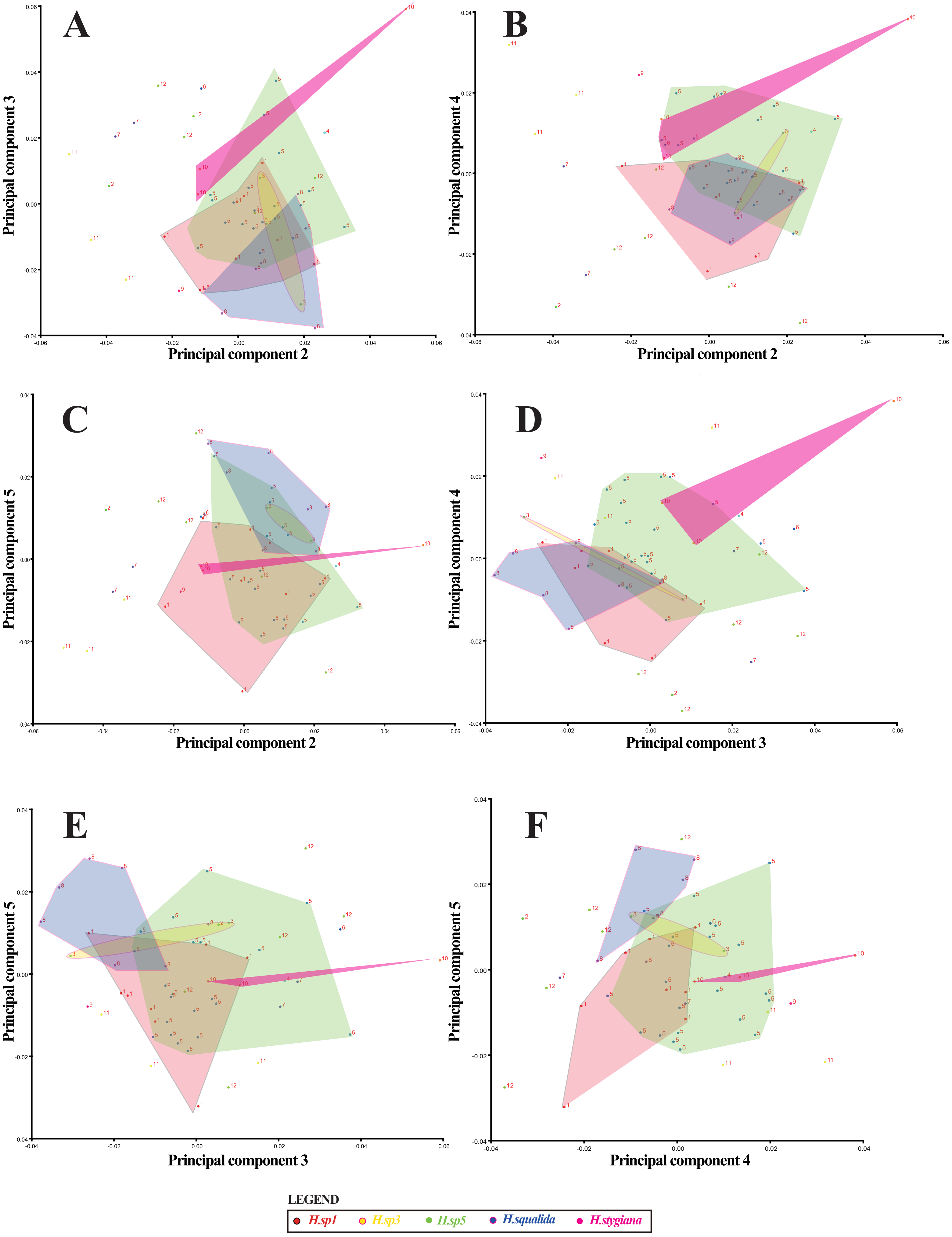
A
Principal component 3
Principal component 2
B
Principal component 4
Principal component 2
C
Principal component 5
Principal component 2
D
Principal component 4
Principal component 3
E
Principal component 5
Principal component 3
F
Principal component 5
Principal component 4
LEGEND
H.sp1
H.sp3
H.sp5
H.squalida
H.stygiana
