# Supplementary material for: A two-step DNA barcoding approach for delimiting moth species: moths of Dongling Mountain (Beijing, China) as a case study
Source: Sci Rep. 2018 Sep 24;8:14256. doi: 10.1038/s41598-018-32123-9 (PMC6155206; doi:10.1038/s41598-018-32123-9)
Supplement: Supplementary file 2 — Supplementary tables [file 41598_2018_32123_MOESM2_ESM.pdf]

A two-step DNA barcoding approach for delimiting moth species: moths of Dongling Mountain (Beijing, China) as a case study

Qian Jin[1†], Xi-Min Hu[2†], Hui-Lin Han[3], Fen Chen[1], Wei-Jia Cai[1], Qian-Qian Ruan[1], Bo Liu[1], Gui-Jie Luo[1], Hao Wang[1], Xu Liu[1], Robert D. Ward[4], Chun-Sheng Wu[5], John-James Wilson[6,7] and Ai-Bing Zhang[2*]

[1]*Suqian Institute of Agricultural Sciences, Jiangsu Academy of Agricultural Sciences, Suqian, Jiangsu 223800*

[2]*College of Life Sciences, Capital Normal University, Beijing 100048, China*

[3]*School of Forestry, Experiment Center, Northeast Forestry University, Haerbin 150040, China*

[4]*CSIRO National Research Collections Australia, GPO Box 1538, Hobart, Tasmania 7001, Australia*

[5]*Institute of Zoology, Chinese Academy of Sciences, 1 Beichen West Road, Chaoyang District, Beijing 100101, China*

[6]*School of Applied Sciences, Faculty of Computing, Engineering and Science, University of South Wales, Pontypridd, CF37 4AT, United Kingdom*

[7]*Department of Microbiology and Parasitology, Faculty of Medical Science, Naresuan University, Phitsanulok 65000, Thailand*

*Corresponding: E-mail: zhangab2008@gmail.com/zhangab2008@mail.cnu.edu.cn

†these authors contributed equally to this work

TableS1 Results of BIN analysis

OTU	Mean	Max	Count	NN Dist
**OTU-1**	0	0	2	6.937394
**OTU-2**	0	0	1	6.937394
**OTU-3**	0	0	2	11.67513
**OTU-4**	0.253807	0.507614	4	8.967851
**OTU-5**	0	0	1	8.967851
**OTU-6**	0.128918	0.338409	7	9.644671
**OTU-7**	0	0	1	9.98308
**OTU-8**	0	0	1	9.137056
**OTU-9**	0	0	1	8.291032
**OTU-10**	0	0	1	5.752961
**OTU-11**	0	0	2	9.137056
**OTU-12**	0	0	1	6.937394
**OTU-13**	0.048344	0.169205	7	6.937394
**OTU-14**	0	0	3	5.752961
**OTU-15**	0.338409	0.507614	3	5.414552
**OTU-16**	0	0	1	7.952622
**OTU-17**	0	0	1	5.414552
**OTU-18**	0.044625	0.169205	14	8.793456
**OTU-19**	0	0	1	6.260575
**OTU-20**	0.305431	1.015228	43	5.583756
**OTU-21**	0	0	1	9.306261
**OTU-22**	0	0	1	5.076142
**OTU-23**	0	0	3	5.076142
**OTU-24**	0.101523	0.169205	5	6.598984
**OTU-25**	0.197406	0.338409	4	6.598984
**OTU-26**	0	0	1	6.091371
**OTU-27**	0	0	2	6.768189
**OTU-28**	0.180485	0.338409	10	10.15228
**OTU-29**	0	0	1	6.091371
**OTU-30**	0.084602	0.169205	4	5.414552
**OTU-31**	0.225606	0.338409	3	7.275804
**OTU-32**	0.225606	0.338409	3	8.291032
**OTU-33**	0.128918	0.338409	7	8.629441
**OTU-34**	0	0	8	5.414552
**OTU-35**	0.966005	1.692047	11	10.15228
**OTU-36**	0	0	1	8.967851
**OTU-37**	0	0	1	8.967851
**OTU-38**	0	0	1	9.644671
**OTU-39**	0	0	1	10.15228
**OTU-40**	0	0	1	7.952622
**OTU-41**	0.203046	0.507614	5	10.49069

**OTU-42**	0	0	1	9.98308
**OTU-43**	0	0	1	8.291032
**OTU-44**	0.277979	0.507614	8	2.368866
**OTU-45**	0	0	1	7.275804
**OTU-46**	0.338409	0.338409	2	7.783418
**OTU-47**	0	0	1	7.614213
**OTU-48**	0.846024	0.846024	2	7.783418
**OTU-49**	0	0	4	2.368866
**OTU-50**	0	0	1	7.275804
**OTU-51**	0	0	1	6.598984
**OTU-52**	0.564016	0.846024	3	6.937394
**OTU-53**	0.084602	0.169205	4	6.937394
**OTU-54**	0	0	1	9.644671
**OTU-55**	0	0	1	8.460238
**OTU-56**	0	0	1	9.644671
**OTU-57**	0.338409	0.338409	2	9.644671
**OTU-58**	0.042301	0.169205	8	8.629441
**OTU-59**	0.564016	0.676819	3	6.598984
**OTU-60**	0	0	1	10.6599
**OTU-61**	0	0	1	8.460238
**OTU-62**	0	0	1	7.783418
**OTU-63**	0	0	1	7.275804
**OTU-64**	0	0	4	11.67513
**OTU-65**	0	0	2	7.952622
**OTU-66**	0.846024	0.846024	2	8.291032
**OTU-67**	0	0	3	9.306261
**OTU-68**	0	0	1	10.99831
**OTU-69**	0	0	1	9.475465
**OTU-70**	0.090243	0.169205	6	9.475465
**OTU-71**	0.136551	0.507614	19	10.6599
**OTU-72**	0	0	1	3.384095
**OTU-73**	0	0	1	3.384095
**OTU-74**	0	0	1	8.798646
**OTU-75**	0.148256	0.507614	15	5.245347
**OTU-76**	0	0	1	7.275804
**OTU-77**	0.112803	0.169205	3	8.121827
**OTU-78**	0	0	4	6.952965
**OTU-79**	0	0	1	3.384095
**OTU-80**	0.73322	2.199662	6	7.614213
**OTU-81**	0	0	1	5.752961
**OTU-82**	0	0	1	8.460238
**OTU-83**	0	0	1	7.614213
**OTU-84**	0	0	2	8.629441
**OTU-85**	0	0	1	5.245347

**OTU-86**	0	0	1	8.967851
**OTU-87**	0	0	1	8.629441
**OTU-88**	0	0	1	7.614213
**OTU-89**	0	0	1	8.460238
**OTU-90**	0.084602	0.169205	4	8.798646
**OTU-91**	0	0	2	6.260575
**OTU-92**	0	0	3	7.445008
**OTU-93**	0	0	2	7.275804
**OTU-94**	0.146886	0.338409	4	6.952965
**OTU-95**	0	0	1	7.614213
**OTU-96**	0	0	1	9.306261
**OTU-97**	0	0	1	8.798646
**OTU-98**	0	0	1	10.49069
**OTU-99**	0	0	1	8.967851
**OTU-100**	0	0	2	8.629441
**OTU-101**	0	0	1	10.32149
**OTU-102**	0	0	1	9.98308
**OTU-103**	0	0	1	11.50592
**OTU-104**	0.338409	0.338409	2	9.98308
**OTU-105**	0	0	2	8.460238
**OTU-106**	0	0	1	9.644671
**OTU-107**	0	0	1	8.798646
**OTU-108**	0	0	1	8.460238
**OTU-109**	0	0	1	8.798646
**OTU-110**	0	0	2	8.798646
**OTU-111**	0	0	1	9.137056
**OTU-112**	0.203046	0.507614	5	5.922166
**OTU-113**	0	0	3	7.106599
**OTU-114**	0	0	2	8.460238

TableS2 Intra-and interspecific divergences of different sequences

Genes	Type of distance	Mean	Min	Max	Std	Number	Number of Min Intert< Max Intra	Rate (%)
CO1	Intra-distance	0.0026	0	0.0102	0.0026	277	642	53.15
	Inter-distance	0.0367	0	0.0975	0.0364	1208		
CO2	Intra-distance	0.0021	0	0.0080	0.0021	277	642	53.15
	Inter-distance	0.0319	0	0.0935	0.0334	1208		
28S	Intra-distance	0.0013	0	0.0051	0.0012	277	689	57.04
	Inter-distance	0.0058	0	0.0207	0.0055	1208		
EF-1a	Intra-distance	0.0017	0	0.0401	0.0046	277	1134	93.87
	Inter-distance	0.0133	0	0.0490	0.0168	1208		
Wgl	Intra-distance	0.0034	0	0.0268	0.0035	277	742	61.42
	Inter-distance	0.0140	0	0.0579	0.0153	1208		
mtDNA	Intra-distance	0.0024	0	0.0083	0.0023	277	642	53.15
	Inter-distance	0.0348	0	0.0935	0.0351	1208		
nDNA	Intra-distance	0.0019	0	0.0197	0.0022	277	866	71.69
	Inter-distance	0.0100	0	0.0357	0.0103	1208		
5 genes combined	Intra-distance	0.0021	0	0.0126	0.0016	277	642	53.15
	Inter-distance	0.0186	0	0.0506	0.0185	1208		

TableS3 Posterior probabilities for different data sets without rjMCMC

Nodes of the species tree	Posterior probabilities (P=tau<0.0002) for different data sets		
	5 loci	n DNA P	mt DNA
spnode20	0.00000	0.00000	0.24708
spnode21	0.00000	0.00000	0.27282
spnode22	0.00000	0.00000	0.31399
spnode23	0.00316	0.02559	0.59826
spnode24	0.73903	0.74681	0.97577
spnode25	0.00000	0.00000	0.42048
spnode26	1.00000	0.99446	0.98885
spnode27	1.00000	0.99977	0.99719
spnode28	1.00000	1.00000	0.99940
spnode29	1.00000	0.99999	0.99975
spnode30	1.00000	0.99810	0.99706
spnode31	1.00000	0.99961	0.99991
spnode32	0.00000	0.00000	0.29423
spnode33	0.00000	0.00000	0.37015
spnode34	0.00003	0.00037	0.53012
spnode35	0.00130	0.00864	0.72495
spnode36	0.03880	0.13163	0.91070
spnode37	0.02289	0.07010	0.75172

TableS4 Taxon information, detailed sampling sites and GenBank accession numbers.

Family	Subfamily	*Species*	Specimen ID	CO1	Locality
Crambidae	Pyraustinae	*Anania verbascalis*	DLS100714092	KJ183366	DLS
Crambidae	Pyraustinae	*Anania verbascalis*	DLS100714336	KJ183367	DLS
Crambidae	Pyraustinae	*Circobotys heterogenalis*	DLS100714158	KJ183368	DLS
Crambidae	Pyraustinae	*Pseudebulea fentoni*	DLS100714130	KJ183369	DLS
Crambidae	Pyraustinae	*Pseudebulea fentoni*	DLS100714287	KJ183370	DLS
Crambidae	Pyraustinae	*Pyrausta sp*	DLS100714140	KJ183371	DLS
Crambidae	Pyraustinae	*Pyrausta sp*	DLS100714213	KJ183372	DLS
Crambidae	Pyraustinae	*Pyrausta sp*	DLS100714342	KJ183373	DLS
Crambidae	Pyraustinae	*Pyrausta sp*	DLS100714350	KJ183374	DLS
Crambidae	Pyraustinae	*Sitochroa verticalis*	DLS100714347	KJ183375	DLS
Crambidae	Spilomelinae	*Diaphania quadrimaculalis*	DLS100714075	KJ183376	DLS
Crambidae	Spilomelinae	*Diaphania quadrimaculalis*	DLS100714167	KJ183377	DLS
Crambidae	Spilomelinae	*Diaphania quadrimaculalis*	DLS100714177	KJ183378	DLS
Crambidae	Spilomelinae	*Diaphania quadrimaculalis*	DLS100714233	KJ183379	DLS
Crambidae	Spilomelinae	*Diaphania quadrimaculalis*	DLS100714302	KJ183380	DLS
Crambidae	Spilomelinae	*Diaphania quadrimaculalis*	DLS100714346	KJ183381	DLS
Crambidae	Spilomelinae	*Diaphania quadrimaculalis*	DLS100714229	KJ183382	DLS
Crambidae	Spilomelinae	*Mecyna flavalis*	DLS100714227	KJ183383	DLS
Erebidae	Arctiinae	*Chionarctia nivea*	DLS100714127	KJ183387	DLS
Erebidae	Arctiinae	*Eilema ussurica*	DLS100714335	KJ183388	DLS
Erebidae	Arctiinae	*Epatolmis caesarea*	DLS100714156	KJ183389	DLS
Erebidae	Arctiinae	*Miltochrista striata*	DLS100714093	KJ183390	DLS
Erebidae	Arctiinae	*Miltochrista striata*	DLS100714321	KJ183391	DLS
Erebidae	Arctiinae	*Pericallia matronula*	DLS100714055	KJ183392	DLS
Erebidae	Arctiinae	*Rhyparioides amurensis*	DLS100714049	KJ183393	DLS
Erebidae	Arctiinae	*Rhyparioides amurensis*	DLS100714050	KJ183394	DLS
Erebidae	Arctiinae	*Rhyparioides amurensis*	DLS100714051	KJ183395	DLS
Erebidae	Arctiinae	*Rhyparioides amurensis*	DLS100714052	KJ183396	DLS
Erebidae	Arctiinae	*Rhyparioides amurensis*	DLS100714053	KJ183397	DLS
Erebidae	Arctiinae	*Rhyparioides amurensis*	DLS100714054	KJ183398	DLS
Erebidae	Arctiinae	*Rhyparioides amurensis*	DLS100714243	KJ183399	DLS
Erebidae	Arctiinae	*Spilosoma lubricipeda*	DLS100714060	KJ183400	DLS
Erebidae	Arctiinae	*Spilosoma lubricipeda*	DLS100714095	KJ183401	DLS
Erebidae	Arctiinae	*Spilosoma lubricipeda*	DLS100714305	KJ183402	DLS
Erebidae	Arctiinae	*Stigmatophora micans*	DLS100714218	KJ183405	DLS
Erebidae	Arctiinae	*Stigmatophora micans*	DLS100714319	KJ183403	DLS
Erebidae	Arctiinae	*Stigmatophora micans*	DLS100714329	KJ183404	DLS
Erebidae	Arctiinae	*Stigmatophora rhodophila*	DLS100714352	KJ183406	DLS
Erebidae	Arctiinae	*Asura unipuncta megala*	DLS100714067	KJ183407	DLS
Erebidae	Lymantrinae	*Euproctis niphonis*	DLS100714139	KJ183488	DLS
Erebidae	Lymantrinae	*Euproctis niphonis*	DLS100714157	KJ183489	DLS

**Erebidae**	**Lymantrinae**	***Euproctis niphonis***	**DLS100714193**	**KJ183490**	**DLS**
**Erebidae**	**Lymantrinae**	***Euproctis niphonis***	**DLS100714194**	**KJ183491**	**DLS**
**Erebidae**	**Lymantrinae**	***Euproctis niphonis***	**DLS100714198**	**KJ183492**	**DLS**
**Erebidae**	**Lymantrinae**	***Euproctis niphonis***	**DLS100714220**	**KJ183493**	**DLS**
**Erebidae**	**Lymantrinae**	***Euproctis niphonis***	**DLS100714222**	**KJ183494**	**DLS**
**Erebidae**	**Lymantrinae**	***Euproctis niphonis***	**DLS100714252**	**KJ183495**	**DLS**
**Erebidae**	**Lymantrinae**	***Euproctis niphonis***	**DLS100714284**	**KJ183496**	**DLS**
**Erebidae**	**Lymantrinae**	***Euproctis niphonis***	**DLS100714292**	**KJ183497**	**DLS**
**Erebidae**	**Lymantrinae**	***Euproctis niphonis***	**DLS100714308**	**KJ183498**	**DLS**
**Erebidae**	**Lymantrinae**	***Euproctis niphonis***	**DLS100714309**	**KJ183499**	**DLS**
**Erebidae**	**Lymantrinae**	***Euproctis niphonis***	**DLS100714315**	**KJ183500**	**DLS**
**Erebidae**	**Lymantrinae**	***Euproctis niphonis***	**DLS100714340**	**KJ183501**	**DLS**
**Erebidae**	**Lymantrinae**	***Dasychira tristis***	**DLS100714113**	**MH122657**	**DLS**
**Erebidae**	**Erebinae**	***Mocis ancilla***	**DLS100714196**	**KJ183426**	**DLS**
**Erebidae**	**Hypeninae**	***Hypena sp.1***	**DLS100714062**	**KF797525**	**DLS**
**Erebidae**	**Hypeninae**	***Hypena sp.1***	**DLS100714076**	**KF797526**	**DLS**
**Erebidae**	**Hypeninae**	***Hypena sp.1***	**DLS100714079**	**KF797527**	**DLS**
**Erebidae**	**Hypeninae**	***Hypena sp.1***	**DLS100714081**	**KF797528**	**DLS**
**Erebidae**	**Hypeninae**	***Hypena sp.1***	**DLS100714089**	**KF797529**	**DLS**
**Erebidae**	**Hypeninae**	***Hypena sp.1***	**DLS100714096**	**KF797530**	**DLS**
**Erebidae**	**Hypeninae**	***Hypena sp.1***	**DLS100714105**	**KF797531**	**DLS**
**Erebidae**	**Hypeninae**	***Hypena sp.1***	**DLS100714110**	**KF797532**	**DLS**
**Erebidae**	**Hypeninae**	***Hypena sp.2***	**DLS100714111**	**KF797533**	**DLS**
**Erebidae**	**Hypeninae**	***Hypena sp.3***	**DLS100714117**	**KF797534**	**DLS**
**Erebidae**	**Hypeninae**	***Hypena sp.3***	**DLS100714131**	**KF797535**	**DLS**
**Erebidae**	**Hypeninae**	***Hypena sp.4***	**DLS100714123**	**KF797536**	**DLS**
**Erebidae**	**Hypeninae**	***Hypena sp.5***	**DLS100714128**	**KF797537**	**DLS**
**Erebidae**	**Hypeninae**	***Hypena sp.5***	**DLS100714144**	**KF797538**	**DLS**
**Erebidae**	**Hypeninae**	***Hypena sp.5***	**DLS100714154**	**KF797539**	**DLS**
**Erebidae**	**Hypeninae**	***Hypena sp.5***	**DLS100714169**	**KF797540**	**DLS**
**Erebidae**	**Hypeninae**	***Hypena sp.5***	**DLS100714179**	**KF797541**	**DLS**
**Erebidae**	**Hypeninae**	***Hypena sp.5***	**DLS100714207**	**KF797542**	**DLS**
**Erebidae**	**Hypeninae**	***Hypena sp.5***	**DLS100714208**	**KF797543**	**DLS**
**Erebidae**	**Hypeninae**	***Hypena sp.5***	**DLS100714214**	**KF797544**	**DLS**
**Erebidae**	**Hypeninae**	***Hypena sp.5***	**DLS100714231**	**KF797545**	**DLS**
**Erebidae**	**Hypeninae**	***Hypena sp.5***	**DLS100714244**	**KF797546**	**DLS**
**Erebidae**	**Hypeninae**	***Hypena sp.5***	**DLS100714247**	**KF797547**	**DLS**
**Erebidae**	**Hypeninae**	***Hypena sp.5***	**DLS100714253**	**KF797548**	**DLS**
**Erebidae**	**Hypeninae**	***Hypena sp.5***	**DLS100714257**	**KF797549**	**DLS**
**Erebidae**	**Hypeninae**	***Hypena sp.5***	**DLS100714261**	**KF797550**	**DLS**
**Erebidae**	**Hypeninae**	***Hypena sp.5***	**DLS100714266**	**KF797551**	**DLS**
**Erebidae**	**Hypeninae**	***Hypena sp.5***	**DLS100714279**	**KF797552**	**DLS**
**Erebidae**	**Hypeninae**	***Hypena sp.5***	**DLS100714281**	**KF797553**	**DLS**
**Erebidae**	**Hypeninae**	***Hypena sp.5***	**DLS100714314**	**KF797554**	**DLS**

**Erebidae**	**Hypeninae**	***Hypena sp.5***	**DLS100714328**	**KF797555**	**DLS**
**Erebidae**	**Hypeninae**	***Hypena sp.5***	**DLS100714351**	**KF797556**	**DLS**
**Erebidae**	**Hypeninae**	***Hypena sp.5***	**DLS100714181**	**KF797557**	**DLS**
**Erebidae**	**Hypeninae**	***Hypena sp.6***	**DLS100714174**	**KF797558**	**DLS**
**Erebidae**	**Hypeninae**	***Hypena sp.7***	**DLS100714176**	**KF797559**	**DLS**
**Erebidae**	**Hypeninae**	***Hypena sp.7***	**DLS100714260**	**KF797560**	**DLS**
**Erebidae**	**Hypeninae**	***Hypena squalida***	**DLS100714083**	**KF797561**	**DLS**
**Erebidae**	**Hypeninae**	***Hypena squalida***	**DLS100714124**	**KF797562**	**DLS**
**Erebidae**	**Hypeninae**	***Hypena squalida***	**DLS100714148**	**KF797563**	**DLS**
**Erebidae**	**Hypeninae**	***Hypena squalida***	**DLS100714166**	**KF797564**	**DLS**
**Erebidae**	**Hypeninae**	***Hypena squalida***	**DLS100714173**	**KF797565**	**DLS**
**Erebidae**	**Hypeninae**	***Hypena squalida***	**DLS100714273**	**KF797566**	**DLS**
**Erebidae**	**Hypeninae**	***Hypena squalida***	**DLS100714334**	**KF797567**	**DLS**
**Erebidae**	**Hypeninae**	***Hypena rivuligera***	**BHS100704018**	**KF797579**	**BHS**
**Erebidae**	**Hypeninae**	***Hypena stygiana***	**BHS100703072**	**KF797576**	**BHS**
**Erebidae**	**Hypeninae**	***Hypena stygiana***	**BHS100703246**	**KF797577**	**BHS**
**Erebidae**	**Hypeninae**	***Hypena stygiana***	**WLS100722081**	**KF797578**	**WLS**
**Erebidae**	**Hypeninae**	***Hypena kengkalis***	**BHS100703096**	**KF797569**	**BHS**
**Erebidae**	**Hypeninae**	***Hypena kengkalis***	**BHS100705124**	**KF797568**	**BHS**
**Erebidae**	**Hypeninae**	***Hypena kengkalis***	**LJZ100813106**	**KF797570**	**LJZ**
**Erebidae**	**Hypeninae**	***Hypena tristalis***	**BHS100704002**	**KF797572**	**BHS**
**Erebidae**	**Hypeninae**	***Hypena tristalis***	**BHS100705023**	**KF797573**	**BHS**
**Erebidae**	**Hypeninae**	***Hypena tristalis***	**BHS100705066**	**KF797574**	**BHS**
**Erebidae**	**Hypeninae**	***Hypena tristalis***	**LJZ100726556**	**KF797575**	**LJZ**
**Erebidae**	**Hypeninae**	***Hypena tristalis***	**WLS100722349**	**KF797571**	**WLS**
**Erebidae**	**Herminiinae**	***Paracolax tristalis***	**DLS100714070**	**KJ183446**	**DLS**
**Erebidae**	**Herminiinae**	***Paracolax tristalis***	**DLS100714232**	**KJ183447**	**DLS**
**Erebidae**	**Herminiinae**	***Paracolax tristalis***	**DLS100714263**	**KJ183448**	**DLS**
**Erebidae**	**Herminiinae**	***Paracolax tristalis***	**DLS100714290**	**KJ183449**	**DLS**
**Erebidae**	**Herminiinae**	***Zanclognatha lunalis***	**DLS100714088**	**KJ183450**	**DLS**
**Erebidae**	**Herminiinae**	***Zanclognatha lunalis***	**DLS100714142**	**KJ183451**	**DLS**
**Erebidae**	**Herminiinae**	***Zanclognatha lunalis***	**DLS100714187**	**KJ183452**	**DLS**
**Erebidae**	**Herminiinae**	***Zanclognatha lunalis***	**DLS100714316**	**KJ183453**	**DLS**
**Erebidae**	**Herminiinae**	***Zanclognatha lunalis***	**DLS100714349**	**KJ183454**	**DLS**
**Erebidae**	**Herminiinae**	***Simplicia sp.***	**DLS100714155**	**KJ183487**	**DLS**
**Erebidae**	**Pangraptinae**	***Pangrapta disruptalis***	**DLS100714091**	**KJ183465**	**DLS**
**Erebidae**	**Pangraptinae**	***Pangrapta disruptalis***	**DLS100714136**	**KJ183466**	**DLS**
**Erebidae**	**Pangraptinae**	***Pangrapta lunulata***	**DLS100714296**	**KJ183467**	**DLS**
**Erebidae**	**Pangraptinae**	***Pangrapta sp.***	**DLS100714164**	**KJ183468**	**DLS**
**Geometridae**	**Ennominae**	***Abraxas sp***	**DLS100714024**	**KJ183225**	**DLS**
**Geometridae**	**Ennominae**	***Abraxas sp***	**DLS100714025**	**KJ183226**	**DLS**
**Geometridae**	**Ennominae**	***Abraxas sp***	**DLS100714026**	**KJ183227**	**DLS**
**Geometridae**	**Ennominae**	***Abraxas sp***	**DLS100714027**	**KJ183228**	**DLS**
**Geometridae**	**Ennominae**	***Abraxas sp***	**DLS100714028**	**KJ183229**	**DLS**

**Geometridae**	**Ennominae**	***Abraxas sp***	**DLS100714029**	**KJ183230**	**DLS**
**Geometridae**	**Ennominae**	***Abraxas sp***	**DLS100714030**	**KJ183231**	**DLS**
**Geometridae**	**Ennominae**	***Abraxas sp***	**DLS100714031**	**KJ183232**	**DLS**
**Geometridae**	**Ennominae**	***Abraxas sp***	**DLS100714032**	**KJ183233**	**DLS**
**Geometridae**	**Ennominae**	***Abraxas sp***	**DLS100714033**	**KJ183234**	**DLS**
**Geometridae**	**Ennominae**	***Amraica confusa***	**DLS100714056**	**KJ183235**	**DLS**
**Geometridae**	**Ennominae**	***Amraica confusa***	**DLS100714074**	**KJ183236**	**DLS**
**Geometridae**	**Ennominae**	***Amraica confusa***	**DLS100714274**	**KJ183237**	**DLS**
**Geometridae**	**Ennominae**	***Arichanna melanariafraterna***	**DLS100714012**	**KJ183238**	**DLS**
**Geometridae**	**Ennominae**	***Arichanna melanariafraterna***	**DLS100714014**	**KJ183239**	**DLS**
**Geometridae**	**Ennominae**	***Arichanna melanariafraterna***	**DLS100714018**	**KJ183240**	**DLS**
**Geometridae**	**Ennominae**	***Arichanna melanariafraterna***	**DLS100714023**	**KJ183241**	**DLS**
**Geometridae**	**Ennominae**	***Arichanna sinica***	**DLS100714013**	**KJ183242**	**DLS**
**Geometridae**	**Ennominae**	***Arichanna sinica***	**DLS100714015**	**KJ183243**	**DLS**
**Geometridae**	**Ennominae**	***Arichanna sinica***	**DLS100714016**	**KJ183244**	**DLS**
**Geometridae**	**Ennominae**	***Arichanna sinica***	**DLS100714017**	**KJ183245**	**DLS**
**Geometridae**	**Ennominae**	***Arichanna sinica***	**DLS100714019**	**KJ183246**	**DLS**
**Geometridae**	**Ennominae**	***Arichanna sinica***	**DLS100714021**	**KJ183248**	**DLS**
**Geometridae**	**Ennominae**	***Arichanna sinica***	**DLS100714022**	**KJ183249**	**DLS**
**Geometridae**	**Ennominae**	***Arichanna sinica***	**DLS100714020**	**KJ183247**	**DLS**
**Geometridae**	**Ennominae**	***Ascotis dianaria***	**DLS100714035**	**KJ183251**	**DLS**
**Geometridae**	**Ennominae**	***Ascotis dianaria***	**DLS100714039**	**KJ183253**	**DLS**
**Geometridae**	**Ennominae**	***Ascotis dianaria***	**DLS100714040**	**KJ183254**	**DLS**
**Geometridae**	**Ennominae**	***Ascotis dianaria***	**DLS100714046**	**KJ183256**	**DLS**
**Geometridae**	**Ennominae**	***Ascotis dianaria***	**DLS100714068**	**KJ183250**	**DLS**
**Geometridae**	**Ennominae**	***Ascotis dianaria***	**DLS100714215**	**KJ183258**	**DLS**
**Geometridae**	**Ennominae**	***Ascotis dianaria***	**DLS100714297**	**KJ183260**	**DLS**
**Geometridae**	**Ennominae**	***Ascotis selenaria***	**DLS100714036**	**KJ183252**	**DLS**
**Geometridae**	**Ennominae**	***Ascotis selenaria***	**DLS100714042**	**KJ183255**	**DLS**
**Geometridae**	**Ennominae**	***Ascotis selenaria***	**DLS100714061**	**KJ183257**	**DLS**
**Geometridae**	**Ennominae**	***Ascotis selenaria***	**DLS100714249**	**KJ183259**	**DLS**
**Geometridae**	**Ennominae**	***Biston betularia***	**DLS100714038**	**KJ183261**	**DLS**
**Geometridae**	**Ennominae**	***Biston betularia***	**DLS100714120**	**KJ183262**	**DLS**
**Geometridae**	**Ennominae**	***Biston betularia***	**DLS100714121**	**KJ183263**	**DLS**
**Geometridae**	**Ennominae**	***Biston thoracicaria***	**DLS100714086**	**KJ183264**	**DLS**
**Geometridae**	**Ennominae**	***Biston thoracicaria***	**DLS100714103**	**KJ183265**	**DLS**
**Geometridae**	**Ennominae**	***Biston thoracicaria***	**DLS100714171**	**KJ183266**	**DLS**
**Geometridae**	**Ennominae**	***Biston thoracicaria***	**DLS100714185**	**KJ183267**	**DLS**
**Geometridae**	**Ennominae**	***Biston thoracicaria***	**DLS100714271**	**KJ183268**	**DLS**
**Geometridae**	**Ennominae**	***Biston thoracicaria***	**DLS100714294**	**KJ183269**	**DLS**
**Geometridae**	**Ennominae**	***Biston thoracicaria***	**DLS100714330**	**KJ183270**	**DLS**
**Geometridae**	**Ennominae**	***Cabera sp***	**DLS100714224**	**KJ183271**	**DLS**
**Geometridae**	**Ennominae**	***Chiasmia hebesata***	**DLS100714320**	**KJ183273**	**DLS**
**Geometridae**	**Ennominae**	***Ectropis excellens***	**DLS100714235**	**KJ183276**	**DLS**

**Geometridae**	**Ennominae**	***Ennomos quercaria***	**DLS100714293**	**KJ183277**	**DLS**
**Geometridae**	**Ennominae**	***Hypomecis punctinalis***	**DLS100714047**	**KJ183285**	**DLS**
**Geometridae**	**Ennominae**	***Hypomecis sp1***	**DLS100714037**	**KJ183286**	**DLS**
**Geometridae**	**Ennominae**	***Hypomecis sp1***	**DLS100714044**	**KJ183287**	**DLS**
**Geometridae**	**Ennominae**	***Hypomecis sp1***	**DLS100714138**	**KJ183288**	**DLS**
**Geometridae**	**Ennominae**	***Hypomecis sp1***	**DLS100714239**	**KJ183289**	**DLS**
**Geometridae**	**Ennominae**	***Hypomecis sp1***	**DLS100714245**	**KJ183290**	**DLS**
**Geometridae**	**Ennominae**	***Hypomecis sp1***	**DLS100714298**	**KJ183291**	**DLS**
**Geometridae**	**Ennominae**	***Hypomecis sp1***	**DLS100714304**	**KJ183292**	**DLS**
**Geometridae**	**Ennominae**	***Hypomecis sp1***	**DLS100714345**	**KJ183293**	**DLS**
**Geometridae**	**Ennominae**	***Hypomecis sp2***	**DLS100714343**	**KJ183294**	**DLS**
**Geometridae**	**Ennominae**	***Hypoxystis pluviaria***	**DLS100714322**	**KJ183295**	**DLS**
**Geometridae**	**Ennominae**	***Jankowskia athleta***	**DLS100714188**	**KJ183296**	**DLS**
**Geometridae**	**Ennominae**	***Jankowskia sp***	**DLS100714112**	**KJ183297**	**DLS**
**Geometridae**	**Ennominae**	***Jankowskia sp***	**DLS100714151**	**KJ183298**	**DLS**
**Geometridae**	**Ennominae**	***Jankowskia sp***	**DLS100714182**	**KJ183299**	**DLS**
**Geometridae**	**Ennominae**	***Jankowskia sp***	**DLS100714205**	**KJ183300**	**DLS**
**Geometridae**	**Ennominae**	***Ophthalmitis albosignaria***	**DLS100714001**	**KJ183305**	**DLS**
**Geometridae**	**Ennominae**	***Ophthalmitis albosignaria***	**DLS100714002**	**KJ183306**	**DLS**
**Geometridae**	**Ennominae**	***Ophthalmitis albosignaria***	**DLS100714003**	**KJ183307**	**DLS**
**Geometridae**	**Ennominae**	***Ophthalmitis albosignaria***	**DLS100714004**	**KJ183308**	**DLS**
**Geometridae**	**Ennominae**	***Ophthalmitis irrorataria***	**DLS100714041**	**KJ183309**	**DLS**
**Geometridae**	**Ennominae**	***Ophthalmitis irrorataria***	**DLS100714043**	**KJ183310**	**DLS**
**Geometridae**	**Ennominae**	***Ophthalmitis irrorataria***	**DLS100714206**	**KJ183311**	**DLS**
**Geometridae**	**Ennominae**	***Phthonandria emaria***	**DLS100714199**	**KJ183312**	**DLS**
**Geometridae**	**Ennominae**	***Phthonandria emaria***	**DLS100714289**	**KJ183313**	**DLS**
**Geometridae**	**Ennominae**	***Phthonandria emaria***	**DLS100714323**	**KJ183314**	**DLS**
**Geometridae**	**Ennominae**	***Selenia sordidaria***	**DLS100714150**	**KJ183326**	**DLS**
**Geometridae**	**Geometrinae**	***Chlorissa obliterata***	**DLS100714211**	**KJ183274**	**DLS**
**Geometridae**	**Geometrinae**	***Comibaena procumbaria***	**DLS100714338**	**KJ183275**	**DLS**
**Geometridae**	**Geometrinae**	***Hemistola veneta***	**DLS100714048**	**KJ183283**	**DLS**
**Geometridae**	**Geometrinae**	***Hemistola veneta***	**DLS100714209**	**KJ183284**	**DLS**
**Geometridae**	**Geometrinae**	***Maxates sp***	**DLS100714085**	**KJ183301**	**DLS**
**Geometridae**	**Geometrinae**	***Maxates sp***	**DLS100714282**	**KJ183302**	**DLS**
**Geometridae**	**Geometrinae**	***Victoria sp***	**DLS100714285**	**KJ183327**	**DLS**
**Geometridae**	**Larentiinae**	***Chartographa sp***	**DLS100714268**	**KJ183272**	**DLS**
**Geometridae**	**Larentiinae**	***Eupithecia abietariagigantean***	**DLS100714119**	**KJ183278**	**DLS**
**Geometridae**	**Larentiinae**	***Eupithecia abietariagigantean***	**DLS100714203**	**KJ183279**	**DLS**
**Geometridae**	**Larentiinae**	***Eupithecia abietariagigantean***	**DLS100714212**	**KJ183280**	**DLS**
**Geometridae**	**Larentiinae**	***Eupithecia abietariagigantean***	**DLS100714226**	**KJ183281**	**DLS**
**Geometridae**	**Larentiinae**	***Eupithecia abietariagigantean***	**DLS100714306**	**KJ183282**	**DLS**
**Geometridae**	**Larentiinae**	***Melanthia sp***	**DLS100714210**	**KJ183303**	**DLS**
**Geometridae**	**Orthostixinae**	***Naxa seriaria***	**DLS100714071**	**KJ183304**	**DLS**
**Geometridae**	**Sterrhinae**	***Problepsis changmei***	**DLS100714005**	**KJ183315**	**DLS**

**Geometridae**	**Sterrhinae**	***Problepsis changmei***	**DLS100714006**	**KJ183316**	**DLS**
**Geometridae**	**Sterrhinae**	***Problepsis changmei***	**DLS100714007**	**KJ183317**	**DLS**
**Geometridae**	**Sterrhinae**	***Problepsis changmei***	**DLS100714008**	**KJ183318**	**DLS**
**Geometridae**	**Sterrhinae**	***Problepsis changmei***	**DLS100714009**	**KJ183319**	**DLS**
**Geometridae**	**Sterrhinae**	***Problepsis changmei***	**DLS100714010**	**KJ183320**	**DLS**
**Geometridae**	**Sterrhinae**	***Problepsis changmei***	**DLS100714011**	**KJ183321**	**DLS**
**Geometridae**	**Sterrhinae**	***Problepsis changmei***	**DLS100714122**	**KJ183322**	**DLS**
**Geometridae**	**Sterrhinae**	***Scopula subpunctaria***	**DLS100714278**	**KJ183323**	**DLS**
**Geometridae**	**Sterrhinae**	***Scopula subpunctaria***	**DLS100714307**	**KJ183324**	**DLS**
**Geometridae**	**Sterrhinae**	***Scopula sp1***	**DLS100714202**	**KJ183325**	**DLS**
**Geometridae**	**UNKNOW**	***Iridopsis_larvaria***	**DLS100714280**	**MH122658**	**DLS**
**Lasiocampidae**	**Pinarinae**	***Gastropacha sikkima***	**DLS100714267**	**KJ183386**	**DLS**
**Limacodidae**	**UNKNOW**	***Chalcoscelides castaneipars***	**DLS100714098**	**KJ183328**	**DLS**
**Limacodidae**	**Limacodinae**	***Monema flavescens***	**DLS100714264**	**KJ183329**	**DLS**
**Limacodidae**	**Limacodinae**	***Narosoideus flavidorsalis***	**DLS100714170**	**KJ183330**	**DLS**
**Limacodidae**	**Limacodinae**	***Narosoideus flavidorsalis***	**DLS100714242**	**KJ183331**	**DLS**
**Limacodidae**	**Limacodinae**	***Narosoideus flavidorsalis***	**DLS100714272**	**KJ183332**	**DLS**
**Limacodidae**	**Limacodinae**	***Narosoideus flavidorsalis***	**DLS100714276**	**KJ183333**	**DLS**
**Limacodidae**	**Limacodinae**	***Narosoideus flavidorsalis***	**DLS100714310**	**KJ183334**	**DLS**
**Limacodidae**	**Limacodinae**	***Narosoideus flavidorsalis***	**DLS100714333**	**KJ183335**	**DLS**
**Limacodidae**	**Limacodinae**	***Parasa consocia***	**DLS100714059**	**KJ183336**	**DLS**
**Limacodidae**	**Limacodinae**	***Parasa consocia***	**DLS100714063**	**KJ183337**	**DLS**
**Limacodidae**	**Limacodinae**	***Parasa consocia***	**DLS100714064**	**KJ183338**	**DLS**
**Limacodidae**	**Limacodinae**	***Parasa consocia***	**DLS100714100**	**KJ183339**	**DLS**
**Limacodidae**	**Limacodinae**	***Parasa consocia***	**DLS100714106**	**KJ183340**	**DLS**
**Limacodidae**	**Limacodinae**	***Parasa consocia***	**DLS100714143**	**KJ183341**	**DLS**
**Limacodidae**	**Limacodinae**	***Parasa consocia***	**DLS100714161**	**KJ183342**	**DLS**
**Limacodidae**	**Limacodinae**	***Parasa consocia***	**DLS100714172**	**KJ183343**	**DLS**
**Limacodidae**	**Limacodinae**	***Parasa consocia***	**DLS100714184**	**KJ183344**	**DLS**
**Limacodidae**	**Limacodinae**	***Parasa consocia***	**DLS100714186**	**KJ183345**	**DLS**
**Limacodidae**	**Limacodinae**	***Parasa consocia***	**DLS100714219**	**KJ183346**	**DLS**
**Limacodidae**	**Limacodinae**	***Parasa consocia***	**DLS100714223**	**KJ183347**	**DLS**
**Limacodidae**	**Limacodinae**	***Parasa consocia***	**DLS100714241**	**KJ183348**	**DLS**
**Limacodidae**	**Limacodinae**	***Parasa consocia***	**DLS100714270**	**KJ183349**	**DLS**
**Limacodidae**	**Limacodinae**	***Parasa consocia***	**DLS100714286**	**KJ183350**	**DLS**
**Limacodidae**	**Limacodinae**	***Parasa consocia***	**DLS100714313**	**KJ183351**	**DLS**
**Limacodidae**	**Limacodinae**	***Parasa consocia***	**DLS100714324**	**KJ183352**	**DLS**
**Limacodidae**	**Limacodinae**	***Parasa consocia***	**DLS100714325**	**KJ183353**	**DLS**
**Limacodidae**	**Limacodinae**	***Parasa consocia***	**DLS100714348**	**KJ183354**	**DLS**
**Limacodidae**	**Limacodinae**	***Thosea sinensis***	**DLS100714114**	**KJ183355**	**DLS**
**Limacodidae**	**Limacodinae**	***Thosea sinensis***	**DLS100714116**	**KJ183356**	**DLS**
**Limacodidae**	**Limacodinae**	***Thosea sinensis***	**DLS100714135**	**KJ183357**	**DLS**
**Limacodidae**	**Limacodinae**	***Thosea sinensis***	**DLS100714149**	**KJ183358**	**DLS**
**Noctuidae**	**Acronictinae**	***Acronicta rumicis***	**DLS100714254**	**KJ183408**	**DLS**

**Noctuidae**	**Acronictinae**	***Acronicta rumicis***	**DLS100714258**	**KJ183409**	**DLS**
**Noctuidae**	**Acronictinae**	***Acronicta intermedia***	**DLS100714318**	**KJ183411**	**DLS**
**Noctuidae**	**Acronictinae**	***Acronicta intermedia***	**DLS100714160**	**KJ183410**	**DLS**
**Noctuidae**	**Acronictinae**	***Acronicta major***	**DLS100714084**	**KJ183412**	**DLS**
**Noctuidae**	**Acronictinae**	***Acronicta major***	**DLS100714250**	**KJ183413**	**DLS**
**Noctuidae**	**Acronictinae**	***Acronicta major***	**DLS100714301**	**KJ183414**	**DLS**
**Noctuidae**	**Amphipyrinae**	***Prospalta cyclica***	**DLS100714080**	**KJ183482**	**DLS**
**Noctuidae**	**Amphipyrinae**	***Prospalta cyclica***	**DLS100714125**	**KJ183483**	**DLS**
**Noctuidae**	**Amphipyrinae**	***Prospalta cyclica***	**DLS100714129**	**KJ183484**	**DLS**
**Noctuidae**	**Amphipyrinae**	***Prospalta cyclica***	**DLS100714341**	**KJ183485**	**DLS**
**Noctuidae**	**Bagisarinae**	***Sphragifera sigillata***	**DLS100714034**	**KJ183415**	**DLS**
**Noctuidae**	**Catocalinae**	***Catocala fulminea***	**DLS100714082**	**KJ183416**	**DLS**
**Noctuidae**	**Catocalinae**	***Catocala fulminea***	**DLS100714104**	**KJ183417**	**DLS**
**Noctuidae**	**Catocalinae**	***Catocala fulminea***	**DLS100714311**	**KJ183418**	**DLS**
**Noctuidae**	**Hadeninae**	***Acosmetia chinensis***	**DLS100714087**	**KJ183422**	**DLS**
**Noctuidae**	**Cuculliinae**	***Cucullia fraudatrix***	**DLS100714137**	**KJ183423**	**DLS**
**Noctuidae**	**Cuculliinae**	***Cucullia fuchsiana***	**DLS100714248**	**KJ183424**	**DLS**
**Noctuidae**	**Cuculliinae**	***Cucullia umbratica***	**DLS100714192**	**KJ183425**	**DLS**
**Noctuidae**	**Hadeninae**	***Acosmetia biguttula***	**DLS100714078**	**KJ183419**	**DLS**
**Noctuidae**	**Hadeninae**	***Acosmetia biguttula***	**DLS100714145**	**KJ183420**	**DLS**
**Noctuidae**	**Hadeninae**	***Acosmetia biguttula***	**DLS100714291**	**KJ183421**	**DLS**
**Noctuidae**	**Hadeninae**	***Lacanobia aliena***	**DLS100714069**	**KJ183427**	**DLS**
**Noctuidae**	**Hadeninae**	***Lacanobia aliena***	**DLS100714099**	**KJ183428**	**DLS**
**Noctuidae**	**Hadeninae**	***Lacanobia aliena***	**DLS100714101**	**KJ183429**	**DLS**
**Noctuidae**	**Hadeninae**	***Lacanobia aliena***	**DLS100714132**	**KJ183430**	**DLS**
**Noctuidae**	**Hadeninae**	***Lacanobia aliena***	**DLS100714133**	**KJ183431**	**DLS**
**Noctuidae**	**Hadeninae**	***Lacanobia aliena***	**DLS100714141**	**KJ183432**	**DLS**
**Noctuidae**	**Hadeninae**	***Lacanobia aliena***	**DLS100714146**	**KJ183433**	**DLS**
**Noctuidae**	**Hadeninae**	***Lacanobia aliena***	**DLS100714163**	**KJ183434**	**DLS**
**Noctuidae**	**Hadeninae**	***Lacanobia aliena***	**DLS100714189**	**KJ183435**	**DLS**
**Noctuidae**	**Hadeninae**	***Lacanobia aliena***	**DLS100714191**	**KJ183436**	**DLS**
**Noctuidae**	**Hadeninae**	***Lacanobia aliena***	**DLS100714195**	**KJ183437**	**DLS**
**Noctuidae**	**Hadeninae**	***Lacanobia aliena***	**DLS100714228**	**KJ183438**	**DLS**
**Noctuidae**	**Hadeninae**	***Lacanobia aliena***	**DLS100714230**	**KJ183439**	**DLS**
**Noctuidae**	**Hadeninae**	***Lacanobia aliena***	**DLS100714238**	**KJ183440**	**DLS**
**Noctuidae**	**Hadeninae**	***Lacanobia aliena***	**DLS100714327**	**KJ183441**	**DLS**
**Noctuidae**	**Hadeninae**	***Sideridis sp***	**DLS100714094**	**KJ183442**	**DLS**
**Noctuidae**	**Heliothinae**	***Raphia peustera***	**DLS100714152**	**KJ183443**	**DLS**
**Noctuidae**	**Heliothinae**	***Raphia peustera***	**DLS100714262**	**KJ183444**	**DLS**
**Noctuidae**	**Heliothinae**	***Schinia scutosa***	**DLS100714277**	**KJ183445**	**DLS**
**Noctuidae**	**Unknow**	***Lophomilia polybapta***	**DLS100714115**	**KJ183479**	**DLS**
**Noctuidae**	**Unknow**	***Lophomilia polybapta***	**DLS100714275**	**KJ183480**	**DLS**
**Noctuidae**	**Noctuinae**	***Athetis pallidipennis***	**DLS100714077**	**KJ183455**	**DLS**
**Noctuidae**	**Noctuinae**	***Eugraphe sigma***	**DLS100714057**	**KJ183471**	**DLS**

**Noctuidae**	**Noctuinae**	***Eugraphe sigma***	**DLS100714216**	**KJ183472**	**DLS**
**Noctuidae**	**Noctuinae**	***Eugraphe sigma***	**DLS100714283**	**KJ183473**	**DLS**
**Noctuidae**	**Noctuinae**	***Lasionycta contigua***	**DLS100714090**	**KJ183456**	**DLS**
**Noctuidae**	**Noctuinae**	***Mythimna monticola***	**DLS100714107**	**KJ183457**	**DLS**
**Noctuidae**	**Noctuinae**	***Mythimna monticola***	**DLS100714118**	**KJ183458**	**DLS**
**Noctuidae**	**Noctuinae**	***Mythimna monticola***	**DLS100714134**	**KJ183459**	**DLS**
**Noctuidae**	**Noctuinae**	***Mythimna monticola***	**DLS100714234**	**KJ183460**	**DLS**
**Noctuidae**	**Noctuinae**	***Mythimna monticola***	**DLS100714344**	**KJ183461**	**DLS**
**Noctuidae**	**Noctuinae**	***Mythimna monticola***	**DLS100714236**	**KJ183462**	**DLS**
**Noctuidae**	**Noctuinae**	***Mythimna velutina***	**DLS100714097**	**KJ183463**	**DLS**
**Noctuidae**	**Ophiderinae**	***Chrysorithrum amata***	**DLS100714332**	**KJ183464**	**DLS**
**Noctuidae**	**Ophiderinae**	***Scoliopteryx libatrix***	**DLS100714303**	**KJ183486**	**DLS**
**Noctuidae**	**Thiacidinae**	***Panthauma egregia***	**DLS100714221**	**KJ183481**	**DLS**
**Noctuidae**	**Xyleninae**	***Eucarta arcta***	**DLS100714165**	**KJ183469**	**DLS**
**Noctuidae**	**Xyleninae**	***Eucarta arcta***	**DLS100714337**	**KJ183470**	**DLS**
**Noctuidae**	**Nolinae**	***Iragaodes nobilis***	**DLS100714317**	**KJ183478**	**DLS**
**Noctuidae**	**Psaphidinae**	***Flexivaleria mienshani***	**DLS100714147**	**KJ183474**	**DLS**
**Noctuidae**	**Psaphidinae**	***Flexivaleria mienshani***	**DLS100714180**	**KJ183475**	**DLS**
**Noctuidae**	**Psaphidinae**	***Flexivaleria mienshani***	**DLS100714190**	**KJ183476**	**DLS**
**Noctuidae**	**Psaphidinae**	***Flexivaleria mienshani***	**DLS100714299**	**KJ183477**	**DLS**
**Notodontidae**	**Dicranurinae**	***Wilemanus bidentatus***	**DLS100714073**	**KJ183502**	**DLS**
**Notodontidae**	**Dicranurinae**	***Wilemanus bidentatus***	**DLS100714126**	**KJ183503**	**DLS**
**Notodontidae**	**Dicranurinae**	***Wilemanus bidentatus***	**DLS100714269**	**KJ183504**	**DLS**
**Notodontidae**	**Dicranurinae**	***Wilemanus bidentatus***	**DLS100714256**	**KJ183505**	**DLS**
**Notodontidae**	**Dudusinae**	***Euhampsonia cristata***	**DLS100714109**	**KF797580**	**DLS**
**Notodontidae**	**Dudusinae**	***Euhampsonia splendida***	**DLS100714162**	**KJ183506**	**DLS**
**Notodontidae**	**Heterocampinae**	***Stauropus basalis***	**DLS100714066**	**KF797581**	**DLS**
**Notodontidae**	**Heterocampinae**	***Stauropus basalis***	**DLS100714300**	**KF797582**	**DLS**
**Notodontidae**	**Notodontinae**	***Gluphisia crenata meridionalis***	**DLS100714183**	**KF797583**	**DLS**
**Notodontidae**	**Notodontinae**	***Nerice hoenei***	**DLS100714246**	**KJ183507**	**DLS**
**Notodontidae**	**Phalerinae**	***Rachiades lichenicolor murzini***	**DLS100714237**	**KJ183508**	**DLS**
**Notodontidae**	**Phalerinae**	***Peridea lativitta***	**DLS100714251**	**KF797584**	**DLS**
**Notodontidae**	**Phalerinae**	***Phalera flavescens***	**DLS100714108**	**KF797585**	**DLS**
**Notodontidae**	**Phalerinae**	***Clostera albosig macurtuloides***	**DLS100714072**	**KJ183509**	**DLS**
**Notodontidae**	**Phalerinae**	***Clostera albosig macurtuloides***	**DLS100714312**	**KJ183510**	**DLS**
**Notodontidae**	**Ptilodoninae**	***Hagapteryx mirabilior***	**DLS100714339**	**KF797586**	**DLS**
**Notodontidae**	**Stauropinae**	***Fentonia ocypete***	**DLS100714217**	**KF797587**	**DLS**
**Sphingidae**	**Macroglossinae**	***Ampelophaga rubiginosa***	**DLS100714353**	**KJ183361**	**DLS**
**Sphingidae**	**Macroglossinae**	***Deilephila askoldensis***	**DLS100714065**	**KJ183362**	**DLS**
**Sphingidae**	**Smerinthinae**	***Callambulyx tatarinovi***	**DLS100714265**	**KJ183363**	**DLS**
**Sphingidae**	**Smerinthinae**	***Dolbina paraexacta***	**DLS100714295**	**KJ183364**	**DLS**
**Sphingidae**	**Smerinthinae**	***Dolbina paraexacta***	**DLS100714326**	**KJ183365**	**DLS**
**Thyatiridae**	**Thyatirinae**	***Tethea albicostata***	**DLS100714058**	**KJ183384**	**DLS**
**Thyatiridae**	**Thyatirinae**	***Tethea albicostata***	**DLS100714331**	**KJ183385**	**DLS**

**Thyatiridae**	**Thyatirinae**	***Tethea ocularis***	**DLS100714045**	**KJ183359**	**DLS**
**Thyatiridae**	**Thyatirinae**	***Tethea ocularis***	**DLS100714102**	**KJ183360**	**DLS**

DLS – Dongling mountain, BHS – Baihua mountain, WLS – Wuling mountain, LJZ – Longjiazhuang

TableS5 Taxon information, detailed sampling sites and GenBank accession numbers of *Hypena*

Family	Subfamily	Species	Specimen ID	COI	COII	28S	EF1-a	Wgl	Locality
**Outgroup**									
Notodontidae	Dudusinae	Euhampsonia cristata	DLS100714109	KF797580	KF797643	KF797517	KF797706	KF797769	DLS
Notodontidae	Heterocampinae	Stauropus basalis	DLS100714066	KF797581	KF797644	KF797518	KF797707	KF797770	DLS
Notodontidae	Heterocampinae	Stauropus basalis	DLS100714300	KF797582	KF797645	KF797519	KF797708	KF797771	DLS
Notodontidae	Notodontinae	Gluphisia crenata meridionalis	DLS100714183	KF797583	KF797646	KF797520	KF797709	KF797772	DLS
Notodontidae	Phalerinae	Peridea lativitta	DLS100714251	KF797584	KF797647	KF797521	KF797710	KF797773	DLS
Notodontidae	Phalerinae	Phalera flavescens	DLS100714108	KF797585	KF797648	KF797522	KF797711	KF797774	DLS
Notodontidae	Ptilodoninae	Hagapteryx mirabilior	DLS100714339	KF797586	KF797649	KF797523	KF797712	KF797775	DLS
Notodontidae	Stauropinae	Fentonia ocypete	DLS100714217	KF797587	KF797650	KF797524	KF797713	KF797776	DLS
**Ingroup**									
Noctuidae	Hypeninae	Hypena sp.1	DLS100714062	KF797525	KF797588	KF797462	KF797651	KF797714	DLS
Noctuidae	Hypeninae	Hypena sp.1	DLS100714076	KF797526	KF797589	KF797463	KF797652	KF797715	DLS
Noctuidae	Hypeninae	Hypena sp.1	DLS100714079	KF797527	KF797590	KF797464	KF797653	KF797716	DLS
Noctuidae	Hypeninae	Hypena sp.1	DLS100714081	KF797528	KF797591	KF797465	KF797654	KF797717	DLS
Noctuidae	Hypeninae	Hypena sp.1	DLS100714089	KF797529	KF797592	KF797466	KF797655	KF797718	DLS
Noctuidae	Hypeninae	Hypena sp.1	DLS100714096	KF797530	KF797593	KF797467	KF797656	KF797719	DLS
Noctuidae	Hypeninae	Hypena sp.1	DLS100714105	KF797531	KF797594	KF797468	KF797657	KF797720	DLS
Noctuidae	Hypeninae	Hypena sp.1	DLS100714110	KF797532	KF797595	KF797469	KF797658	KF797721	DLS
Noctuidae	Hypeninae	Hypena sp.2	DLS100714111	KF797533	KF797596	KF797470	KF797659	KF797722	DLS
Noctuidae	Hypeninae	Hypena sp.3	DLS100714117	KF797534	KF797597	KF797471	KF797660	KF797723	DLS
Noctuidae	Hypeninae	Hypena sp.3	DLS100714131	KF797535	KF797598	KF797472	KF797661	KF797724	DLS
Noctuidae	Hypeninae	Hypena sp.4	DLS100714123	KF797536	KF797599	KF797473	KF797662	KF797725	DLS
Noctuidae	Hypeninae	Hypena sp.5	DLS100714128	KF797537	KF797600	KF797474	KF797663	KF797726	DLS
Noctuidae	Hypeninae	Hypena sp.5	DLS100714144	KF797538	KF797601	KF797475	KF797664	KF797727	DLS
Noctuidae	Hypeninae	Hypena sp.5	DLS100714154	KF797539	KF797602	KF797476	KF797665	KF797728	DLS
Noctuidae	Hypeninae	Hypena sp.5	DLS100714169	KF797540	KF797603	KF797477	KF797666	KF797729	DLS
Noctuidae	Hypeninae	Hypena sp.5	DLS100714179	KF797541	KF797604	KF797478	KF797667	KF797730	DLS
Noctuidae	Hypeninae	Hypena sp.5	DLS100714181	KF797542	KF797605	KF797479	KF797668	KF797731	DLS
Noctuidae	Hypeninae	Hypena sp.5	DLS100714207	KF797543	KF797606	KF797480	KF797669	KF797732	DLS
Noctuidae	Hypeninae	Hypena sp.5	DLS100714208	KF797544	KF797607	KF797481	KF797670	KF797733	DLS
Noctuidae	Hypeninae	Hypena sp.5	DLS100714214	KF797545	KF797608	KF797482	KF797671	KF797734	DLS
Noctuidae	Hypeninae	Hypena sp.5	DLS100714231	KF797546	KF797609	KF797483	KF797672	KF797735	DLS
Noctuidae	Hypeninae	Hypena sp.5	DLS100714244	KF797547	KF797610	KF797484	KF797673	KF797736	DLS
Noctuidae	Hypeninae	Hypena sp.5	DLS100714247	KF797548	KF797611	KF797485	KF797674	KF797737	DLS

**Noctuidae**	**Hypeninae**	**Hypena sp.5**	**DLS100714253**	**KF797549**	**KF797612**	**KF797486**	**KF797675**	**KF797738**	**DLS**
**Noctuidae**	**Hypeninae**	**Hypena sp.5**	**DLS100714257**	**KF797550**	**KF797613**	**KF797487**	**KF797676**	**KF797739**	**DLS**
**Noctuidae**	**Hypeninae**	**Hypena sp.5**	**DLS100714261**	**KF797551**	**KF797614**	**KF797488**	**KF797677**	**KF797740**	**DLS**
**Noctuidae**	**Hypeninae**	**Hypena sp.5**	**DLS100714266**	**KF797552**	**KF797615**	**KF797489**	**KF797678**	**KF797741**	**DLS**
**Noctuidae**	**Hypeninae**	**Hypena sp.5**	**DLS100714279**	**KF797553**	**KF797616**	**KF797490**	**KF797679**	**KF797742**	**DLS**
**Noctuidae**	**Hypeninae**	**Hypena sp.5**	**DLS100714281**	**KF797554**	**KF797617**	**KF797491**	**KF797680**	**KF797743**	**DLS**
**Noctuidae**	**Hypeninae**	**Hypena sp.5**	**DLS100714314**	**KF797555**	**KF797618**	**KF797492**	**KF797681**	**KF797744**	**DLS**
**Noctuidae**	**Hypeninae**	**Hypena sp.5**	**DLS100714328**	**KF797556**	**KF797619**	**KF797493**	**KF797682**	**KF797745**	**DLS**
**Noctuidae**	**Hypeninae**	**Hypena sp.5**	**DLS100714351**	**KF797557**	**KF797620**	**KF797494**	**KF797683**	**KF797746**	**DLS**
**Noctuidae**	**Hypeninae**	**Hypena sp.6**	**DLS100714174**	**KF797558**	**KF797621**	**KF797495**	**KF797684**	**KF797747**	**DLS**
**Noctuidae**	**Hypeninae**	**Hypena sp.7**	**DLS100714176**	**KF797559**	**KF797622**	**KF797496**	**KF797685**	**KF797748**	**DLS**
**Noctuidae**	**Hypeninae**	**Hypena sp.7**	**DLS100714260**	**KF797560**	**KF797623**	**KF797497**	**KF797686**	**KF797749**	**DLS**
**Noctuidae**	**Hypeninae**	**Hypena squalida**	**DLS100714083**	**KF797561**	**KF797624**	**KF797498**	**KF797687**	**KF797750**	**DLS**
**Noctuidae**	**Hypeninae**	**Hypena squalida**	**DLS100714124**	**KF797562**	**KF797625**	**KF797499**	**KF797688**	**KF797751**	**DLS**
**Noctuidae**	**Hypeninae**	**Hypena squalida**	**DLS100714148**	**KF797563**	**KF797626**	**KF797500**	**KF797689**	**KF797752**	**DLS**
**Noctuidae**	**Hypeninae**	**Hypena squalida**	**DLS100714166**	**KF797564**	**KF797627**	**KF797501**	**KF797690**	**KF797753**	**DLS**
**Noctuidae**	**Hypeninae**	**Hypena squalida**	**DLS100714173**	**KF797565**	**KF797628**	**KF797502**	**KF797691**	**KF797754**	**DLS**
**Noctuidae**	**Hypeninae**	**Hypena squalida**	**DLS100714273**	**KF797566**	**KF797629**	**KF797503**	**KF797692**	**KF797755**	**DLS**
**Noctuidae**	**Hypeninae**	**Hypena squalida**	**DLS100714334**	**KF797567**	**KF797630**	**KF797504**	**KF797693**	**KF797756**	**DLS**
**Noctuidae**	**Hypeninae**	**Hypena rivuligera**	**BHS100704018**	**KF797579**	**KF797642**	**KF797505**	**KF797704**	**KF797768**	**BHS**
**Noctuidae**	**Hypeninae**	**Hypena stygiana**	**BHS100703072**	**KF797576**	**KF797639**	**KF797506**	**KF797702**	**KF797765**	**BHS**
**Noctuidae**	**Hypeninae**	**Hypena stygiana**	**BHS100703246**	**KF797577**	**KF797640**	**KF797507**	**KF797703**	**KF797766**	**BHS**
**Noctuidae**	**Hypeninae**	**Hypena stygiana**	**WLS100722081**	**KF797578**	**KF797641**	**KF797508**	**KF797705**	**KF797767**	**WLS**
**Noctuidae**	**Hypeninae**	**Hypena kengkalis**	**BHS100703096**	**KF797569**	**KF797632**	**KF797509**	**KF797695**	**KF797758**	**BHS**
**Noctuidae**	**Hypeninae**	**Hypena kengkalis**	**BHS100705124**	**KF797568**	**KF797631**	**KF797510**	**KF797694**	**KF797757**	**BHS**
**Noctuidae**	**Hypeninae**	**Hypena kengkalis**	**LJZ100813106**	**KF797570**	**KF797633**	**KF797511**	**KF797696**	**KF797759**	**LJZ**
**Noctuidae**	**Hypeninae**	**Hypena tristalis**	**BHS100704002**	**KF797572**	**KF797635**	**KF797512**	**KF797698**	**KF797761**	**BHS**
**Noctuidae**	**Hypeninae**	**Hypena tristalis**	**BHS100705023**	**KF797573**	**KF797636**	**KF797513**	**KF797699**	**KF797762**	**BHS**
**Noctuidae**	**Hypeninae**	**Hypena tristalis**	**BHS100705066**	**KF797574**	**KF797637**	**KF797514**	**KF797700**	**KF797763**	**BHS**
**Noctuidae**	**Hypeninae**	**Hypena tristalis**	**LJZ100726556**	**KF797575**	**KF797638**	**KF797515**	**KF797701**	**KF797764**	**LJZ**
**Noctuidae**	**Hypeninae**	**Hypena tristalis**	**WLS100722349**	**KF797571**	**KF797634**	**KF797516**	**KF797697**	**KF797760**	**WLS**

DLS – Dongling mountain, BHS – Baihua mountain, WLS – Wuling mountain, LJZ – Longjiazhuang
