# Supplementary material for: A two-step DNA barcoding approach for delimiting moth species: moths of Dongling Mountain (Beijing, China) as a case study
Source: Sci Rep. 2018 Sep 24;8:14256. doi: 10.1038/s41598-018-32123-9 (PMC6155206; doi:10.1038/s41598-018-32123-9)
Supplement: Supplementary file 3 — Appendix1–2 [file 41598_2018_32123_MOESM3_ESM.pdf]

A two-step DNA barcoding approach for delimiting moth species: moths of Dongling Mountain (Beijing, China) as a case study

Qian Jin[1†], Xi-Min Hu[2†], Hui-Lin Han[3], Fen Chen[1], Wei-Jia Cai[1], Qian-Qian Ruan[1], Bo Liu[1], Gui-Jie Luo[1], Hao Wang[1], Xu Liu[1], Robert D. Ward[4], Chun-Sheng Wu[5], John-James Wilson[6,7] and Ai-Bing Zhang[2*]

[1]*Suqian Institute of Agricultural Sciences, Jiangsu Academy of Agricultural Sciences, Suqian, Jiangsu 223800*

[2]*College of Life Sciences, Capital Normal University, Beijing 100048, China*

[3]*School of Forestry, Experiment Center, Northeast Forestry University, Haerbin 150040, China*

[4]*CSIRO National Research Collections Australia, GPO Box 1538, Hobart, Tasmania 7001, Australia*

[5]*Institute of Zoology, Chinese Academy of Sciences, 1 Beichen West Road, Chaoyang District, Beijing 100101, China*

[6]*School of Applied Sciences, Faculty of Computing, Engineering and Science, University of South Wales, Pontypridd, CF37 4AT, United Kingdom*

[7]*Department of Microbiology and Parasitology, Faculty of Medical Science, Naresuan University, Phitsanulok 65000, Thailand*

*Corresponding: E-mail: zhangab2008@gmail.com/zhangab2008@mail.cnu.edu.cn

†these authors contributed equally to this work

Appendix 1 Detailed description of morphospecies

H. kengkalis

Adult. Wingspan: 28-30mm. Head: grayish brown. Thorax and abdomen: grayish brown. Forewing: darker grayish brown; antemedial line brown, outward oblique from costal margin to posterior part of cell, then inward oblique to inner margin; median line very indistinct; postmedial line brown, inward oblique from costal margin to inner margin, curved at M_3; subterminal line formed by black dots; terminal line grayish yellow; orbicular spot only a small, brown dot; reniform spot indistinct and oblate. Hindwing: dark gray, spread brown; discal spot indistinct, brown; grayish yellow fringe.

Male genitalia. Uncus: sickle shaped, rather slender. Tegumen: twice as long as uncus. Valva: sandal shaped, saccular margin with a curve at the middle; clasper similar triangle shape; costal flat and straight; separate line extends to the top of the 1/3 length. Juxta: triangle jar shaped. Saccus: U-shaped. Aedeagus: with a sclerotized carina and finger shaped; vesica with a cornuti plate at base, with a central grain plate.

H. riv

Adult. Wingspan: 29-31mm. Head: yellowish brown. Thorax and abdomen: brown, yellowish. Forewing: brown; antemedial and median lines very indistinct; postmedial line light brown, inward oblique from costal margin to inner margin, curved at M_2; subterminal line grey and slender; terminal line dark brown; orbicular spot indistinct; reniform spot black, indistinct and cashew shaped; a white line from basal part of costa outward oblique to postmedial line in inner margin area, and light grey inside, and blackish brown outside to postmedial line where it forms a big spot; subterminal and terminal line areas dark brown. Hindwing: dark gray; discal spot indistinct, brown; subterminal line light grey and slender.

Female genitalia. Papillae analis: short and cone shaped, slightly sclerotized.

Apophysis posterioris: 4/5 as long as apophysis anterioris. Antrum: U shaped. Ductus bursae: membranous, slender at middle and posterioris part, rather gradually thickening at anterioris part. Corpus bursae: long oval shaped, membranous, with very small grain; with a curved at posterior part.

***H. sp.*1**

Adult. Wingspan: 30-31mm. Head: yellowish grey. Thorax and abdomen: blackish brown. Forewing: dark blackish brown, orange diffuse; antemedial and median lines very indistinct; postmedial line light grayish white, inward oblique from costal margin to inner margin, curved at M_2; subterminal line grayish white and very slender; terminal line dark, yellowish brown; orbicular and reniform spots indistinct; a grayish white line from base of costa outward oblique to inner margin, terminates about 1/3from the base part, and with postmedial line forming a big black spot; subterminal and terminal lines areas dark brown. Hindwing: dark smoky black; discal spot indistinct, black; fringe with some orange.

Male genitalia. Uncus: hook shaped, rather short and thick, slightly sclerotized. Tegumen: 1.5 times as long as uncus. Valva: oval shaped, saccular margin straight; clasper short right angle process and sclerotized; separate line about 2/5 length of valva and broad; costa thick, broad, with fold at top. Juxta: sclerotized, lung shaped. Saccus: U shaped and broad. Aedeagus: hockey stick shaped; carina with cornuti band; vesica with a big cornutus at the base.

***H. sp.*2**

Adult. Wingspan: 30-32mm. Head, thorax and abdomen: grayish black to black, thorax diffuse brown. Forewing: dark blackish brown to smoky black, diffuse a little orange; antemedial line dark black, appears at costal margin, but otherwise indistinct; median lines very indistinct; postmedial line thin grayish white, inward oblique from costal margin to inner margin, curved at M_2 and Cu_2; subterminal line very thin grayish white; terminal line thin yellowish brown; orbicular spot indistinct; reniform spot a small black dot, with thin white outer frame; subterminal and terminal lines

areas brownish black; postmedial line to base dark blackish brown. Hindwing: dark smoky black; discal spot indistinct, black; terminal line black; fringe same ground color.

Male genitalia. Uncus: hook shaped, rather short and thick, slightly sclerotized. Tegumen: 2.5 times as long as uncus. Valva: oval shaped, saccular margin slightly sunken near the base; clasper short acute angle process and sclerotized; separate line 2/5 length of valva and broad; costa rather thick, broad, flat, and smooth at top; processus of costa larg and extend to nearby the saccular margin. Juxta: sclerotized, triangle shaped. Saccus: U shaped and broad. Aedeagus: smooth and slightly curved; vesica with a big cornutus at middle part, and two small cornuti plates.

***H. sp.* 3**

Adult. Wingspan: 32-34mm. Head: grayish brown. Thorax and abdomen: blackish brown. Forewing: dark brownish black to smoky black; antemedial line dark brown, outward oblique to the middle part of inner margin; median line very indistinct; postmedial line thin grayish white, inward oblique from costal margin to inner margin, curved at M_{2-3} and 2A, strongly inward oblique in inner margin area; subterminal line very thin grey; terminal line thin black; orbicular spot indistinct; reniform spot black, indistinct; terminal lines area light black; subterminal line area rather darker than terminal lines area; postmedial line to base dark black. Hindwing: dark brownish black, diffuse orange; discal spot and cross lines disappear.

Male genitalia. Uncus: hook shaped, rather short and thick, slightly sclerotized, very sharp at top part. Tegumen: 2.25 times as long as uncus. Valva: sandal shaped, saccular margin slightly sunken at the middle; clasper short acute angle process and sclerotized; separate line about 1/3 length of valva and broad; costa rather thick, broad, flat, with fold near the top; processus of costa rather narrow and extend to the center part of valva; basal part straight, and broader than top. Juxta: sclerotized, alcohol lamp shaped. Saccus: U-shaped and broad. Aedeagus: curved at middle part, caecum slightly swell; carina sclerotized, with short cornuti band, and a long pine shaped cornutus at basal part; vesica with a big cornuti plate at the base, and a small cornuti

plate.

_H. sp._ 4

Wingspan: 30-31mm. The adult of this species is very similar to *H. sp.3* (the wing patterning of *H. sp.4* had been damaged, so cannot be described). These two species can be distinguished in the male genitalia, uncus of *H. sp.3* curved more sharply than *H. sp.4*; tegument of *H. sp.3* 2.25 times as long as uncus, whereas *H. sp.4* tegument is 2 times times as long as uncus; top of valva in *H. sp.3* narrower than *H. sp.4*; the ridge of posterioris part of the costal process in *H. sp.3* extends outward, whereas in *H. sp.4* it is oblique outward; carina triangle, flat, with a long pine shaped cornutus in *H. sp.3*, in *H. sp.4* carina only straight ridge, not flat, absent long cornutus.

_H. sp._ 5

Wingspan: 30-31mm. The adult of this species is very similar to *H. sp.1*, but can be distinguished by the apex area of forewing being reddish brown, and in *H. sp.1* very thin; the postmedial line is slightly curved between M_2 and 2A, in *H. sp.1* it is smooth; hindwing reddish brown, in *H. sp.1* dark smoky black, diffuse very little reddish brown. In male genitalia, uncus long sharp at top and smooth at base, in *H. sp.1* short sharp at top and not smooth at base; clasper acute angle, with broad arc at the outside, in *H. sp.1* right angle, with narrow arc at the outside; the ridge of posterioris part of the costal process is rather straight and oblique outward, in *H. sp.1* thick arc shaped; tegumen about 2.4 times as long as uncus, in *H. sp.1* 1.5 times; the carina rather thin and sharp, in *H. sp.1* thick and blunt.

_H. sp._ 6

Adult. Wingspan: 30-31mm. Head: grayish yellow. Thorax: grayish brown, diffuse light grey. Abdomen: blackish brown. Forewing: dark brownish black to smoky black; antemedial and median lines very indistinct; postmedial line grayish white, from costal margin extend to M2, then form a obtuse angle at $M_{2\text{-}3}$, after this inward oblique to inner margin; subterminal line very thin grayish white; terminal line thin brown; orbicular spot indistinct; reniform spot dark brown, halo; terminal lines area

light grey; subterminal line area rather darker than terminal lines area; postmedial line to base dark black; an outward oblique line grey extends from the middle of the base to the the base of inner margin. Hindwing: dark smoky black, diffuse a little orange; discal spot appear, halo.

Male genitalia. Uncus: hook shaped, strong, sclerotized, very sharp at top part. Tegumen: about 1.6 times as long as uncus. Valva: thin, long oval shaped, saccular margin slightly sunken about 1/3 of the distance to the base; clasper short right angle process and strongly sclerotized; separate line 1/3 length of valva and broad; costa rather broader, flat; processus of costa thin, and about 2/3 length of the width; basal part straight, and narrower than top. Juxta: sclerotized, alcohol lamp shaped. Saccus: narrow U shaped. Aedeagus: curved at middle part; carina sclerotized, blunt ridge, with cornuti band; vesica with a small and sclerotized cornuti plate at the base, and a long and rather thin cornuti band at terminal part.

H. sp. 7

Wingspan: 29-30mm. The adult of this species is very similar to *H. sp.6,* but can be distinguished by the sharp apex, in *H. sp.6* rather blunt; the postmedial line with a obtuse angle, sharper and shorter than *H. sp.6*; orbicular and reniform spots disntinct than *H. sp.6*; between postmedial line and base part blackish brown, in *H. sp.6* black; hindwing grayish brown, in *H. sp.6* smoky black. In male genitalia, uncus rather thin sclerotized, in *H. sp.6* hardly sclerotized; clasper acute angle, with hardly sclerotized, broad arc at the outside, in *H. sp.6* right angle, with narrow arc at the outside; the ridge of posterioris part of costa arc, in *H. sp.6* rather straight; tegumen about 2 times as long as uncus, in *H. sp.6* 1.6 times; the carina big swell, with a short cornuti band, in *H. sp.6* small swell, with a long cornuti band; the posterioris part of aedeagus broad, in *H. sp.6* narrow.

H. squalida

Wingspan: 30mm. The adult of this species is very similar to *H. riv*, but can be distinguished by the blunt apex, in *H. sp.6* rather sharp; the postmedial line with a

curved angle, in *H. riv* rather smooth; between postmedial line and base dark brownish black, in *H. riv* blackish brown; terminal line area smoky black, in *H. riv* brown; hindwing smoky black, in *H. riv* dark gray. In female, corpus bursae short oval shaped, with a signa, and smooth at posterior part, in *H. riv* long oval shaped, without signa, with a curved at posterior part.

H. sty

Wingspan: 29-30mm. The adult of this species is very similar to *H. sp.5*, but can be distinguished by the apex area of forewing being reddish brown, in *H. sp.5* rather sharp ; the postmedial line is slightly curved between M_2 and 2A, in *H. sp.5* smooth; hindwing reddish brown, in *H. sty* dark smoky black. In male genitalia, uncus short sharp at top and not smooth at basal part , in *H. sp5* uncus long sharp at top and smooth at basal part; clasper acute angle.

H. tristalis

Wingspan: 30-32mm. Head: mixed black and orange. Thorax: orange and mixed black; abdomen light grayish brown. Forewing: light grayish brown; antemedial and median lines very indistinct; postmedial line brown and indistinct; subterminal line form by black dots between viens; terminal line form by black dots between viens; costal margin area dark black between base to postmedial line, and swell at terminal part. Hindwing: light grayish brown.

Male genitalia. Uncus: flat and sclerotized, hook shaped, slightly swell at middle part. Tegumen: about 1.6 times as long as uncus. Valva: sandal shaped, clasper small, only slightly bulge; cosa flat, process part lag spread shaped; top part broader than bottom part. Saccus: narrow U shaped. Aedeagus: curved at middle; carina slightly sclerotized, with a small bulge; vesica with two cornuti paltes at base and middle part.

Appendix 2 Detailed tree construction

Maximum Likelihood (ML), Neighbor-Joining (NJ) and Maximum Parsimony (MP) methods were used to construct trees of the 55 *Hypena* specimens. Gene regions were analyzed both separately (COI, COII 28S, EF-1α and Wgl) and in combination (mt+nDNA, nDNA, and mtDNA).

The best fit model of DNA substitution was selected using ModelTest 3.7 (Posada & Crandall 2001), under the Bayesian Information Criterion (Luo *et al.* 2010). The best fit model for 28S, COII, mt+nDNA, nDNA, and mtDNA was GTR+I+G. The best fit model for COI, EF-1α and Wgl, was GTR+G, TN93+I+G and TN93+G, respectively.

ML trees were reconstructed using PhyML version3.0 (Guindon *et al.* 2010). Starting trees were distance based (BIONJ) according to the default option, and nonparametric bootstrap analyses based on 1000 replications were carried out to determine the approximate level of support for all branching events, with support percentages computed by majority-rule consensus. NJ trees were constructed using MEGA 7 based on the K2P (Kimura-2-parameter) distance model. The branch supports were estimated through 1000 bootstrap replications, and other parameters were set to their default settings. For MP trees, bootstrap analyses (Felsenstein 1985, 1988) using MEGA 7, based on a full heuristic search of 1000 replications using SPR, were carried out to determine support levels of each node, and clades with bootstrap values < 50% were collapsed. This method finds multiple MP trees, and we obtained the final MP consensus Tree.
